# A Multifaceted Digital Intervention for the Prevention of Type 2 Diabetes Mellitus in Primary Care (PREDIABETEXT): Cluster Randomized Trial

**DOI:** 10.2196/70981

**Published:** 2025-10-09

**Authors:** Sofía Mira-Martínez, Narges Malih, Escarlata Angullo-Martínez, Rocío Zamanillo-Campos, Aina M Yañez, Miquel Bennasar-Veny, Rocío Gómez-Juanes, Jadwiga Konieczna, Rafael Jiménez, Maria J Serrano-Ripoll, Maria Antonia Fiol-deRoque, Alfonso Leiva, Aina M Galmes-Panades, Jerónima Miralles-Xamena, Maria Clara Vidal-Thomàs, Cristina Gómez-Cobo, Elena Gervilla, José Iván Oña-Gil, Ignacio Ricci-Cabello

**Affiliations:** 1 Health Research Institute of the Balearic Islands Palma Spain; 2 Research Group on Global Health and Human Development University of the Balearic Islands Palma Spain; 3 Red de Investigación en Cronicidad Atención Primaria y Prevención y Promoción de la Salud Madrid Spain; 4 Centro de Investigación Biomédica en Red, Epidemiología y Salud Pública Institute of Health Carlos III Madrid Spain; 5 Department of Medicine University of the Balearic Islands Palma Spain; 6 Centro de Investigación Biomédica en Red, Fisiopatología de la Obesidad y Nutrición Institute of Health Carlos III Madrid Spain; 7 Department of Psychology University of the Balearic Islands Palma Spain; 8 Primary Care Research Unit of Mallorca Balearic Islands Health Service Palma Spain; 9 Physical Activity and Sport Sciences Research Group, Institute for educational research and innovation University of the Balearic Islands Palma Spain; 10 Clinical Analysis Service Hospital Universitario Son Espases Palma Spain; 11 Research Group on Community Nutrition and Oxidative Stress, Research Institute of Health Sciences University of the Balearic Islands Palma Spain; 12 Dra Teresa Piqué Primary Health Care Center Balearic Islands Health Service Palma Spain

**Keywords:** prediabetic state, preventive health services, clinical trial, primary health care, digital health, blood glucose, glycated hemoglobin, SMS text messaging, risk factors

## Abstract

**Background:**

The diabetes epidemic continues to surge worldwide, demanding urgent and innovative solutions. Digital health interventions, particularly those targeting behavior change, hold promise due to their affordability and scalability. However, research in this field remains in its early stages.

**Objective:**

This study aimed to evaluate the effectiveness of PREDIABETEXT (Prediabetes Text Message Digital Intervention for the Prevention of Type 2 Diabetes Mellitus), a digital health intervention, in reducing glycated hemoglobin (HbA_1c_) and in improving secondary clinical, physiological, and behavioral outcomes.

**Methods:**

We conducted a 6-month, 3-arm, pragmatic cluster randomized clinical trial. We recruited patients with prediabetes (HbA_1c_ levels between 6% and 6.4% in the previous 3 months or 2 consecutive fasting plasma glucose measurements of 110-125 mg/dL) registered at primary care centers in the Balearic Islands, Spain. The PREDIABETEXT intervention consisted of 3 personalized SMS text messages per week aimed at supporting lifestyle behavior changes and online training for their primary health care professionals. A total of 58 professionals (clusters) from 16 centers participated in the study and were randomized (1:1:1) to intervention group A (patient SMS text messaging), intervention group B (patient SMS text messaging+health care professional web-based training), or the control group (usual care). Following the 6-month intervention period, we conducted individual qualitative interviews with 8 patients and 7 health care professionals to evaluate their experiences with the intervention in terms of utility, satisfaction, and implementation barriers.

**Results:**

In total, 58 health care professionals (clusters) were included, allocated to the control group (n=20, 34%; 119/365, 32.6% patients), intervention group A (SMS text messaging only; n=18, 31%; 106/365, 29% patients), and intervention group B (SMS text messaging+training; n=20, 34%; 140/365, 38.4% patients). The mean age of the patients was 59.79 (SD 9.75) years, and 54.5% (199/365) were female. The results of the intention-to-treat analysis at the 6-month time point showed that intervention A led to a small, nonsignificant reduction in HbA_1c_ levels compared to the control group (β=−0.05, 95% CI −0.21 to 0.10; *P*=.50), whereas intervention B showed a similar nonsignificant reduction (β=−0.04, 95% CI −0.12 to 0.10; *P*=.56). No substantial differences were observed in the remaining secondary outcomes. Interviews revealed positive feedback from patients, who appreciated the intervention’s dietary messages and their frequency and practicality. Participants suggested enhancements such as increased personalization, links to recipes, and nursing follow-ups. Health care professionals valued the online training but highlighted time constraints, suggesting shorter or blended formats to improve accessibility.

**Conclusions:**

While PREDIABETEXT did not significantly improve HbA_1c_ levels, it demonstrated potential benefits for patient engagement. Further studies involving more intensive interventions are warranted to confirm the clinical impact on diabetes prevention.

**Trial Registration:**

ClinicalTrials.gov NCT05110625; https://www.clinicaltrials.gov/study/NCT05110625

**International Registered Report Identifier (IRRID):**

RR2-10.3390/ijerph192214706

## Introduction

### Background

Prediabetes is a significant global health concern, with a worldwide prevalence of 9.1% (464 million people) based on impaired glucose tolerance in 2021 projected to rise to 10% (638 million people) by 2045 [1]. Similarly, impaired fasting glucose affected 5.8% of the global population (298 million people) in 2021, with an anticipated increase to 6.5% (414 million people) by 2045 [2]. In Spain, the age-adjusted prevalence of impaired glucose tolerance was 6.2% in 2021, affecting 2,600,800 adults, whereas impaired fasting glucose prevalence was 2.8%, impacting 1,231,400 individuals [3].

Lifestyle modifications have proven effective in preventing diabetes [4], particularly among individuals with risk factors such as overweight, a family history of the disease, or physical inactivity [5]. One of the most successful programs, the year-long Diabetes Prevention Program, reduced the incidence of type 2 diabetes mellitus (T2DM) by 58% over 3 years in its randomized controlled trial (RCT) [6]. However, barriers such as time constraints and low commitment often hinder patients with prediabetes from engaging in intensive interventions [7]. Digitally delivered, lower-intensity interventions offer a promising alternative by addressing these barriers [8]. Advances in digital health technologies such as wearables, mobile health (mHealth) apps, and data analytics enable personalized health education and real-time monitoring [9]. These innovations have the potential to enhance chronic disease management, including diabetes and cardiovascular conditions.

While some studies indicate that mHealth interventions may reduce the incidence of T2DM, improve glucose tolerance, promote weight loss, and lower glycated hemoglobin (HbA_1c_) levels [10-12], recent systematic reviews highlight that the field is still in its early stages and the evidence remains inconclusive [13,14]. Moreover, the potential to enhance the effectiveness of mHealth interventions by combining them with educational cointerventions for health care professionals has not yet been explored.

### Objectives

To address this gap, we developed PREDIABETEXT (Prediabetes Text Message Digital Intervention for the Prevention of Type 2 Diabetes Mellitus), a novel, theory-driven, multifaceted digital intervention that contributes to existing research by integrating health care professional training with patient-centered interventions. By combining personalized SMS text messaging for patients with an online training program for health care professionals, our approach aimed to enhance diabetes prevention by supporting patient lifestyle changes while equipping health care professionals with the skills to effectively manage prediabetes in primary care.

Therefore, this study aimed to evaluate the effects of PREDIABETEXT on HbA_1c_ levels (primary outcome) and additional clinical, behavioral, and psychological outcomes through a 3-arm, 6-month cluster RCT.

## Methods

### Overview

The trial protocol is available elsewhere [15]. This trial was prospectively registered at ClinicalTrials.gov (NCT05110625) on November 8, 2021, before participant recruitment. The reporting of this study is based on the CONSORT (Consolidated Standards of Reporting Trials) 2010 statement extension to cluster RCTs [16] (Multimedia Appendix 1).

### Trial Design

This study consisted of a pragmatic, 6-month, 3-arm cluster RCT conducted in primary care centers in Mallorca (Balearic Islands, Spain). Clusters were defined at the level of health care professionals working in these centers to account for variability in individual practice styles and patient interactions. Among the 58 health care professionals included, 16 centers were represented, with a median of 3 (IQR 1-6) health care professionals per center. To minimize contamination, access to the online training (intervention group B) was restricted through password protection. Randomization at the health care professional level ensured that practice-level contamination was unlikely as control health care professionals did not receive the training. This design helped maintain intervention fidelity at the health care professionals level while limiting the spread of intervention content to control health care professionals even within the same practice.

### Participants

#### Eligibility Criteria for the Clusters

Physicians and nurses from primary health care centers in Mallorca were invited to participate in the study via email, which included detailed information about the study. Participation was confirmed through follow-up phone calls, during which participant information were collected and informed consent was audio recorded. Health care professionals with a higher number of patients meeting the prediabetes criteria were prioritized for inclusion. Professionals who were planning to transfer to another primary health care unit during the study period were excluded.

#### Eligibility Criteria for the Patients

Patients were eligible for inclusion if they were aged between 18 and 75 years; registered with the Balearic Islands Health Service; and at risk of T2DM based on at least one of the following criteria: HbA_1c_ level between 6% and 6.4% in the previous 3 months, 2 consecutive fasting plasma glucose (FPG) values between 110 and 125 mg/dL, or both. In addition, participants were required to have access to a mobile device capable of receiving SMS text messages. Patients were excluded if they were unable to read messages in Spanish, had a severe mental health condition, were currently using antidiabetic medication, had given birth in the previous 12 months or were pregnant, or were planning to change health care centers during the intervention period.

### Interventions

#### Overview

The PREDIABETEXT intervention was designed at the cluster level targeting both health care professionals and their patients. It was developed in accordance with the Medical Research Council guidelines for complex interventions [17] and was informed by the extended normalization process theory [18] based on the findings of a formative qualitative study involving both patients and primary care providers [19]. This approach ensured that the intervention was aligned with the needs of the target population and feasible to implement within the health care system. A detailed description of the PREDIABETEXT intervention is available elsewhere [15], whereas its development is outlined in this section.

A multidisciplinary team comprising primary care physicians and nurses, endocrinologists, nutritionists, sports scientists, psychologists, and pharmacists developed the PREDIABETEXT cointervention for patients. This intervention included up to 500 SMS text messages based on various behavior change techniques to promote engagement and facilitate sustainable lifestyle changes among participants [20]. It targeted individuals at risk of T2DM and was refined through a series of iterative workshops. To ensure that the content was clear and relevant, 3 patients actively participated in the development process. In addition, 2 diabetes experts reviewed the prefinal draft to ensure accuracy and alignment with current clinical guidelines. The intervention was delivered via SMS text messages using the Balearic Islands Health Service messaging system.

To assess the intervention’s acceptability, relevance, and perceived impact, it was piloted for 1 month with 21 individuals with prediabetes. A total of 12 patients from diverse socioeconomic backgrounds participated in individual semistructured interviews after the pilot trial to explore their experiences and challenges using the intervention. The intervention was subsequently refined based on these findings.

The cointervention for health care professionals was developed by a multidisciplinary team of endocrinologists, nutritionists, physicians, nurses, and primary care education managers in the Balearic Islands. The online training was designed to address health care professionals’ educational needs, preferences, and perceived barriers to implementation as identified through interviews with 15 physicians and nurses from diverse settings and with varying levels of knowledge about prediabetes. To refine the intervention, a pilot study was conducted with 10 primary care physicians and nurses, who reviewed the materials and completed a questionnaire on their usefulness and suggested improvements. Feedback from the pilot was incorporated to enhance the training content. This approach ensured that the intervention was both relevant to health care professionals’ needs and feasible for implementation in the primary care setting.

#### Intervention Group A: Patients Receiving Text Messages

The PREDIABETEXT intervention included SMS text messages, each 160 characters long, addressing key topics to promote behavior change toward a healthier lifestyle. These messages were sent 3 times per week over a 6-month period. The messages focused primarily on nutrition and physical activity. Regarding nutrition, recommendations were made regarding the types of carbohydrates that help prevent glycemic spikes; appropriate portion sizes; the frequency of food group consumption; and guidance on limiting ultraprocessed food, alcohol, and energy drink consumption. The messages also included recipe examples. Regarding physical activity, participants received advice on the frequency, intensity, and duration of exercise, as well as techniques to reduce sedentary behavior and strategies to replace sedentary activities with more physically active ones. In addition, participants received motivational messages and personalized content targeting weight loss or smoking cessation, if applicable. All messages fit one of the behavior change techniques according to the taxonomy proposed by Michie et al [20]. The patients’ PREDIABETEXT cointervention is reported according to the Template for Intervention Description and Replication checklist [21] in Multimedia Appendix 2. Multimedia Appendix 3 contains representative examples of the SMS text messages used in the intervention for the patients.

#### Intervention Group B: Patients Receiving Text Messages and Health Care Professional Training

In this group, patients received the same SMS text messaging intervention as those in intervention group A. In addition, health care professionals participated in a 16-hour accredited online training program titled *Clinical Management of Prediabetes*, which was awarded 2.4 European Credit Transfer and Accumulation System credits as part of the continuing education program for health professionals of the Balearic Islands Health Service. To prevent contamination bias, access to the course was restricted through password protection, ensuring that only professionals in intervention group B could participate.

The training was structured into 6 teaching units and delivered through a combination of theoretical content, narrated slide presentations, and supplementary materials. Topics included an introduction to prediabetes; epidemiology; diagnostic criteria; behavior modification strategies; treatment approaches (nutrition, physical activity, and pharmacological treatment); and monitoring and follow-up, including management in children and adolescents and the prevention of complications. The course also incorporated specific content on communication techniques to facilitate behavior change and included video demonstrations of clinical consultations.

Professionals were instructed to complete the training within a 1-month period. They were given flexibility to complete the course in a single session or divide it into multiple sessions based on their availability. Progress was tracked via the platform’s learning management system. An initial and final knowledge test was administered to assess learning outcomes, and a satisfaction questionnaire was completed at the end of the program.

#### Control Group: Usual Care

Patients in the control group received usual care, consisting of standard primary care services in accordance with routine clinical practice within the Balearic Islands’ public health care system. This did not include any structured educational materials, targeted counseling, or specific diabetes prevention programs beyond what is typically offered during general consultations. Their health care professionals did not receive the online training intervention. This ensured that any structured behavioral intervention was delivered exclusively to the intervention groups, thereby minimizing the risk of contamination or confounding effects.

### Sample Size

The sample size was estimated based on the primary outcome variable (HbA_1c_ levels). We planned to recruit 42 primary health care professionals and 420 patients (approximately 10 patients per health care professionals). On the basis of an expected SD of 0.8%, an intraclass correlation coefficient of 0.04 [22], a design effect of 1.36, and an anticipated dropout rate of 10%, a total of 420 participants (140 per study arm) was deemed sufficient to achieve 80% power to detect a between-group difference of at least 0.3% in HbA_1c_ levels. This effect size was selected based on previous evidence suggesting that even modest reductions in HbA_1c_ levels are associated with clinically meaningful improvements in diabetes risk and related complications [23]. A meta-analysis of 47 RCTs including 7677 patients demonstrated that self-management interventions resulted in a mean HbA_1c_ level reduction of 0.36% (95% CI 0.21-0.51) [24]. We designed our clinical trial to detect a 0.3% decrease in HbA_1c_ levels, which is a lower threshold than 0.5% as the minimum for clinical importance [25], to make sure that it was sensitive enough to identify even these slight changes.

### Recruitment

#### Recruitment of Health Care Professionals

Eligible health care professionals were invited to participate via email, which included an attached participant information sheet detailing the study.

#### Recruitment of Patients

Patients were recruited from the rosters of health care professionals who had already agreed to participate. Using the information service platform (see the Acknowledgments section), a list of eligible patients was compiled from the Balearic Islands primary care database. Each health care professional received a list of approximately 10 eligible patients generated through standardized queries to the electronic health record system. To ensure up-to-date baseline data, patients with the most recent laboratory test results—particularly HbA_1c_ values from the previous 3 months—were prioritized. Patients were then sorted based on the date of their recorded HbA_1c_ values and contacted sequentially following the study’s eligibility criteria until the target number of participants per health care professional (n=10) was reached.

Potential participants received an SMS text message invitation through the Balearic Islands Health Service’s SMS text messaging system. After 48 hours, a research assistant followed up with a phone call to formally invite patients to the study, provide additional information, and obtain informed consent via phone. Further details regarding the recruitment procedure are provided in Multimedia Appendix 4.

### Data Collection

#### Health Care Professional Data

An online ad hoc questionnaire assessing knowledge about the management of prediabetes was administered at baseline and at the end of the course for professionals in intervention group B and to all participating professionals 6 months after completing the intervention.

#### Patient Data

After enrollment, baseline data on participants’ lifestyle behaviors were collected through semistructured phone interviews. Subsequently, participants attended an in-person visit with a research nurse either at their primary care center or at the reference hospital depending on their preference. During this visit, anthropometric measurements were taken, and blood samples were collected. Recruitment and follow-up of participants were conducted sequentially.

### Randomization and Blinding

Recruited physicians and nurses were assigned to 1 of 3 study arms using computer-generated random numbers. Their patients were invited to participate in the study and received the intervention according to the allocation of their health care professionals. Allocation concealment was performed at the level of health care professionals (clusters). Once baseline data collection for both health care professionals and patients was completed, the project manager (SM-M) assigned health care professionals (clusters) to intervention groups based on the pregenerated randomization sequence. To maintain allocation concealment, a centralized randomization process was implemented. Health care professionals were blinded to the allocation sequence before patient recruitment, ensuring unbiased assignment of participants. These measures ensured appropriate cluster formation and rigorous randomization, thereby maintaining the integrity of the study design. Notably, the randomization process was not stratified by any specific factors. Details regarding the randomization procedures are provided in Multimedia Appendix 4.

Due to the nature of the intervention, health care professionals and patients could not be blinded to group allocation. However, outcome assessors (ie, questionnaire administrators and laboratory staff) and statisticians were blinded to group assignment.

### Outcomes

#### Primary Outcome

The primary outcome was HbA_1c_ levels at the 6-month time point. HbA_1c_ level was selected as the primary outcome because it is a well-established, objective biomarker that reflects long-term glycemic control and is widely used in diabetes research and clinical practice [26,27]. This choice aligns with the study’s research objective, which was to assess the effectiveness of the PREDIABETEXT digital intervention in preventing T2DM among individuals with prediabetes.

#### Secondary Outcomes

Regarding clinical and physiological outcomes, FPG, triglycerides, total cholesterol, low-density lipoprotein (LDL) cholesterol, high-density lipoprotein (HDL) cholesterol, lipoprotein(a), aspartate aminotransferase, alanine aminotransferase, gamma-glutamyl transpeptidase, complete blood count, serum creatinine, serum albumin, insulin, urinary sediment, and urinary albumin were assessed. To reduce variability, all blood samples were analyzed at a central laboratory using standardized assay methods, ensuring consistency across study sites. Cardiovascular disease risk was evaluated using the Girona Heart Registry (REGICOR)–Framingham risk equation, which classifies individuals aged 35 to 74 years in Spain as moderate or high risk (≥5%) or low risk (<5%) [28]. Insulin resistance was assessed using the homeostatic model assessment [29]. In addition, diabetes incidence was calculated as the proportion of patients newly diagnosed with T2DM. We assessed risk of progression to type 2 diabetes by classifying participants into 3 categories based on baseline glycemic measures: low risk (altered FPG levels only; 100-125 mg/dL), medium risk (altered HbA_1c_ levels only; 6%-6.4%), and high risk (both altered FPG, 100-125 mg/dL and altered HbA_1c_, 6%-6.4% levels). Changes in these risk categories from baseline to 6 months were analyzed.

Motivational outcomes were assessed using a brief motivation questionnaire. Adherence to the Mediterranean diet was measured using the Mediterranean Diet Adherence Screener, with scores of <9 indicating low adherence and scores of ≥9 indicating strong adherence [30]. Physical activity was evaluated using the REGICOR Short Physical Activity Questionnaire and classified into 3 categories: not very active (<300 metabolic equivalent of task [MET] minutes per week), active (600-1200 MET minutes per week), and highly active (>1200 MET minutes per week) [31]. Sedentary behavior was measured using the Spanish version of the Nurses’ Health Study Physical Activity Questionnaire [32]. Smoking habits were assessed through an adapted version of the Global Adult Tobacco Survey and were classified as current smoker or former or never smoker [33], and alcohol consumption was quantified using a validated alcohol intake calculator [34].

Knowledge of prediabetes management among professionals in intervention group B was assessed using a 15-item ad hoc questionnaire. The questionnaire was developed based on current clinical practice guidelines, mainly from the American Diabetes Association and the World Health Organization, covering critical aspects of prediabetes care, including epidemiology, diagnosis, behavioral interventions, pharmacological treatments, cardiovascular risk management, physical activity recommendations, and motivational interviewing techniques. A group of endocrinologists and primary care physicians reviewed the questionnaire to make sure it was clear, clinically relevant, and in line with the most recent guidelines for managing prediabetes. The questionnaire is available in Multimedia Appendix 5. Each correct answer was scored as 1 point (incorrect=0), with total scores ranging from 0 to 15.

### Statistical Methods

Descriptive statistics were used to examine the baseline characteristics of the participants. The effect of the intervention was evaluated by analyzing differences in primary and secondary outcomes between groups at the 6-month time point. All participants received the intervention as randomized, with no protocol deviations. To ensure robustness and avoid attrition bias, we used the intention-to-treat (ITT) approach, which includes all participants as originally allocated regardless of adherence or intervention completion. The missing values, outliers, and invalid data were handled using multiple imputations by chained equations. This process was repeated for 10 iterations, generating multiple imputed datasets. Final imputed values were obtained by averaging those from each iteration. Trace plots were used to assess stability and convergence across iterations. Standardized effect coefficients and their corresponding 95% CIs were computed. Between-group differences in HbA_1c_ levels and other outcomes were analyzed using generalized estimating equations (GEE), which accounted for the correlation between repeated measures within individuals. GEE assumes between-subject independence and within-subject dependence, modeling the within-subject correlation matrix accordingly. This method allowed for the evaluation of time and group effects on intervention outcomes while preserving the ITT framework. The clustering effect was addressed using an appropriate working correlation structure. All statistical analyses were conducted using SPSS (version 25; IBM Corp) and Stata (version 16; StataCorp), with statistical significance set at 5%.

### Within-Trial Process Evaluation Qualitative Study

After completing the intervention, we conducted individual interviews with the participants to explore their experiences with the PREDIABETEXT intervention. We contacted them via phone call, with up to 3 call attempts if they were unanswered. Patient interviews took place after the 6-month intervention period and no later than 1 month from the completion of the intervention.

Health care professionals who received the cointervention were interviewed within a month of completing their training. They were purposefully sampled for diversity in gender, age, and professional category (nurse or physician).

Two dynamic topic guides for patients and professionals (Multimedia Appendix 6) were used, encompassing a global evaluation of the intervention’s utility and satisfaction alongside key themes relevant to the development of digital solutions for chronic disease management. The guide was iteratively refined as data collection progressed. Supportive prompts were used only when necessary to elicit responses aligned with the study objectives. All interviews were conducted via telephone by 2 trained members of the research team (RZ-C and SM-M), both of whom were independent from the participants’ clinical care and had no previous relationship with them. The interviews, lasting approximately 15 to 40 minutes, were audio recorded, transcribed verbatim, and anonymized. Data were collected until thematic saturation was reached, meaning that no new pertinent themes emerged from participants who gave their consent. Data analysis was managed and conducted using the NVivo software (version 12; QSR International), which facilitated coding and theme development in line with the approach by Clarke and Braun [35]. Throughout this study, 2 researchers (SM-M and RZ-C) worked together to analyze the interviews. The researchers critically assessed their own role and the study’s objective to avoid influencing participants’ responses.

### Ethical Considerations

All procedures were approved in June 2021 by the Primary Care Research Committee and the Balearic Islands Research Ethics Committee (reference IB4495/21PI). This study complied with the ethical principles outlined in the Declaration of Helsinki.

To ensure data confidentiality and integrity, all participant information was anonymized before analysis, securely stored on encrypted servers at the Health Research Institute of the Balearic Islands, and accessed only by authorized members of the research team under strict confidentiality agreements. Data protection measures adhered to national and European regulations, including the General Data Protection Regulation.

Consent from the participants was obtained, and they were informed of their right to withdraw at any time. No financial compensation was provided; participation was entirely voluntary. All information collected was treated confidentially and anonymized, and ethical oversight was maintained throughout the study.

## Results

### Participant Flow

**Figure 1:**
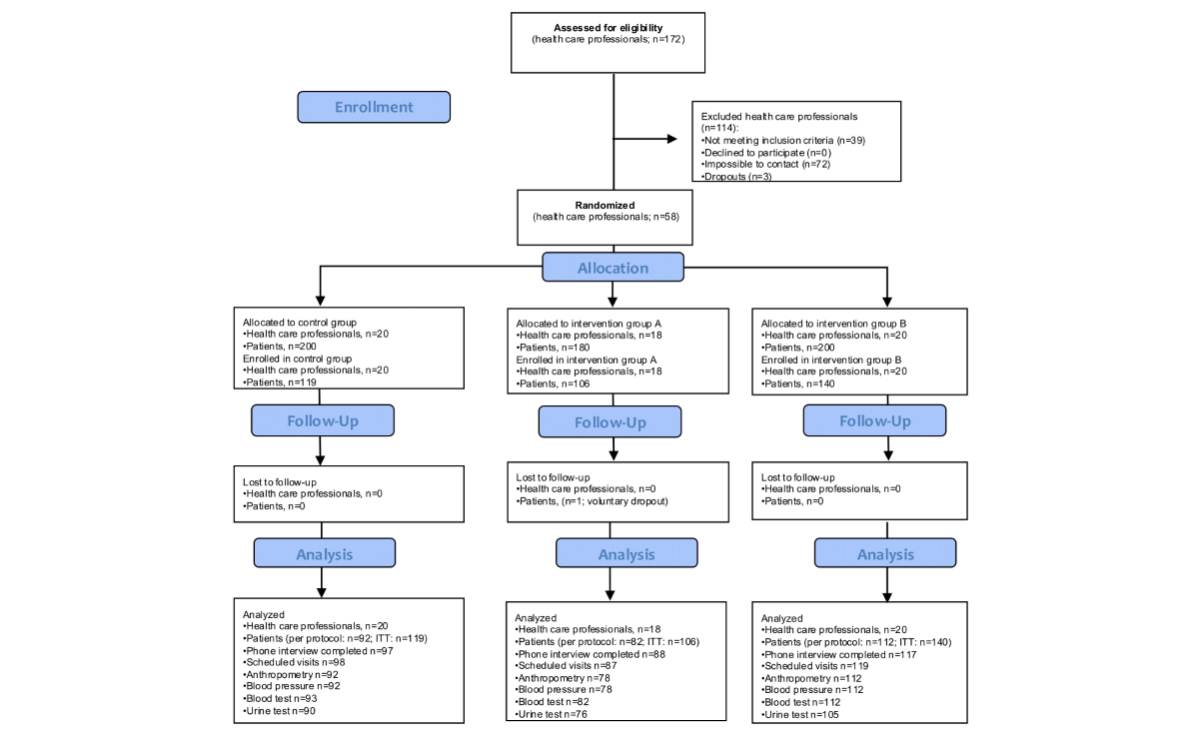
CONSORT flow diagram of the study participants. ITT: intention to treat.

The participants’ flow through the study is illustrated in the CONSORT flowchart in Figure 1. Recruitment of health care professionals began on October 20, 2021, and concluded on June 1, 2022. Of the 172 professionals invited, 58 (33.7%; n=30, 52% physicians and n=28, 48% nurses) from 16 centers consented to participate and completed the baseline telephone interview, yielding a recruitment rate of 43.6% (58/133). Of these 58 professionals, 18 (31%) were allocated to intervention group A, 20 (34%) were allocated to intervention group B, and 20 (34%) were allocated to the control group. Notably, there were no dropouts among health care professionals during the 6-month intervention period.

Patient recruitment took place between November 17, 2021, and August 2, 2022. A total of 365 patients were enrolled. Randomization occurred at the health care professional level, with each health care professional (cluster) assigned to 1 of 3 groups: intervention group A (n=106, 29% of the patients), intervention group B (n=140, 38.4% of the patients), or the control group (n=119, 32.6% of the patients) according to the health care professional’s allocation. Postintervention data were collected at the 6-month time point from 78.4% (286/365) of the participants, resulting in an attrition rate of 21.6% (79/365). The distribution of participants who completed the 6-month period was as follows: 28.7% (82/286) from intervention group A, 39.2% (112/286) from intervention group B, and 32.2% (92/286) from the control group. The primary reasons for attrition included inability to reach participants via phone or failure to attend in-person appointments. Missing values for various outcomes were primarily due to these reasons or because some participants completed the questionnaires but did not attend para-clinical assessments.

### Baseline Characteristics

The health care professionals had a mean age of 49.69 (SD 10.15) years, with most being female (48/58, 83%). On average, participants reported 23.59 (SD 9.22) years of professional experience in health care.

Table 1 presents baseline patient demographics and clinical characteristics summarized at the level of 58 health care professionals (clusters) allocated to the control group (n=20, 34% professionals; 119/365, 32.6% patients), intervention group A (SMS text messaging only; n=18, 31% professionals; 106/365, 29% patients), and intervention group B (SMS text messaging+training; n=20, 34% professionals; 140/365, 38.4% patients). The average percentage of female patients per professional was 42.02% (SD 22.89%) in the control group, 49.06% (SD 23.72%) in intervention group A, and 45.71% (SD 18.05%) in intervention group B. The mean patient age per professional was 60.75 (SD 3.40) years in the control group, 58.66 (SD 2.78) years in intervention group A, and 59.84 (SD 3.83) years in intervention group B, with an overall mean of 59.79 (SD 3.50) years. These results indicate a broadly comparable demographic profile across clusters at baseline, supporting the validity of between-group comparisons in subsequent outcome analyses.

**Table 1:** Baseline demographic and clinical characteristics of clusters by trial arm (N=365).

		Total	Control (n=119)	Intervention group A (SMS text messaging; n=106)	Intervention group B (SMS text messaging+training; n=140)
**Demographic characteristics**					
	Women, n (%)	45.48 (21.54)	42.02 (22.89)	49.06 (23.72)	45.71 (18.05)
	Age (y), mean (SD)	59.79 (3.50)	60.75 (3.40)	58.66 (2.78)	59.84 (3.83)
**Clinical characteristics, mean (SD)**					
	Height (cm)	162.82 (4.23)	162.29 (4.85)	163.28 (3.99)	162.93 (3.81)
	Weight (kg)	84.38 (8.85)	84.71 (12.52)	84.57 (6.49)	83.96 (6.31)
	BMI (kg/m^2^)	31.73 (2.68)	31.98 (3.63)	31.68 (2.13)	31.54 (2.03)
	WC^a^ (cm)	103.77 (5.97)	103.49 (8.11)	103.92 (4.92)	103.89 (4.37)
	HC^b^ (cm)	110.06 (5.14)	110.93 (6.69)	109.35 (4.21)	109.87 (4.09)
	WHR^c^	0.94 (0.03)	0.93 (0.03)	0.95 (0.03)	0.94 (0.03)
	SBP^d^ (mm Hg)	133.32 (6.08)	130.77 (6.10)	135.22 (5.45)	134.05 (5.82)
	DBP^e^ (mm Hg)	76.33 (4.62)	74.56 (4.93)	78.75 (3.27)	76.00 (4.45)
	FPG^f^ (mg/dL)	104.28 (5.53)	102.90 (5.03)	105.18 (5.79)	104.78 (5.55)
	TG^g^ (mg/dL)	141.18 (41.68)	123.48 (18.11)	156.85 (40.66)	144.37 (50.56)
	Cholesterol (mg/dL)	194.69 (12.27)	195.98 (12.51)	195.33 (12.54)	193.10 (11.76)
	LDL^h^ (mg/dL)	117.39 (11.05)	121.49 (9.66)	116.43 (12.54)	114.63 (9.95)
	HDL^i^ (mg/dL)	49.67 (4.48)	50.16 (3.18)	48.58 (5.26)	50.07 (4.66)
	TG/HDL	3.17 (1.79)	2.65 (0.49)	3.65 (1.26)	3.24 (2.56)
	Cholesterol/HDL	3.97 (0.51)	3.98 (0.28)	4.16 (0.41)	3.81 (0.67)
	HbA_1c_^j^ (%)	6.13 (0.06)	6.13 (0.05)	6.11 (0.07)	6.15 (0.05)
	Insulin level (U/mL)	16.94 (8.87)	14.78 (4.63)	19.68 (7.31)	16.52 (11.28)
	HOMA^k^	4.87 (3.67)	3.76 (1.38)	5.11 (2.16)	5.46 (5.13)
	REGICOR-Framingham^l^ score (%)	5.77 (1.23)	5.80 (1.33)	5.63 (1.22)	5.86 (1.15)
**Categories of the REGICOR-Framingham score, n (%)**					
	Low risk	40.27 (20.81)	37.82 (20.72)	50.00 (18.86)	35.00 (19.90)
	Moderate or high risk	57.26 (21.32)	59.66 (22.28)	49.06 (18.58)	61.43 (20.84)
**Adherence to the Mediterranean diet**					
	MEDAS^m^ score, mean (SD)	7.52 (0.77)	7.41 (0.93)	7.56 (0.76)	7.58 (0.59)
	Low adherence, n (%)	67.40 (18.30)	69.75 (18.41)	61.32 (16.85)	70.00 (18.29)
	Good adherence, n (%)	32.60 (18.30)	30.25 (18.41)	38.68 (16.85)	30.00 (18.29)
**Physical activity level**					
	Not very active, n (%)	52.33 (20.45)	57.98 (18.30)	51.89 (23.16)	47.86 (18.90)
	Active, n (%)	22.74 (16.30)	18.49 (10.53)	21.70 (21.07)	27.14 (15.20)
	Very active, n (%)	24.93 (17.79)	23.53 (18.06)	26.42 (18.61)	25.00 (16.93)
	MET^n^ min per wk, mean (SD)	1983.78 (1020.61)	1987.94 (1074.71)	1914.54 (1130.97)	2032.66 (880.32)
**Sedentary lifestyle, mean (SD)**					
	Daily h per d watching television	3.52 (0.75)	3.64 (0.67)	3.48 (1.05)	3.44 (0.49)
	Daily h sitting in front of a computer, mobile, or tablet screen	0.98 (0.58)	1.18 (0.72)	0.92 (0.46)	0.85 (0.49)
	Daily h per d sitting in any means of transportation	0.33 (0.22)	0.27 (0.16)	0.29 (0.23)	0.41 (0.23)
	Daily h per d sitting	4.57 (0.74)	4.84 (0.60)	4.45 (1.03)	4.42 (0.46)
**Smoking habit, n (%)**					
	Former or never smoker	80.27 (15.39)	79.83 (11.72)	87.74 (13.09)	75.00 (17.42)
	Current smoker	19.73 (15.39)	20.17 (11.72)	12.26 (13.09)	25.00 (17.42)
**Alcohol use, mean (SD)**					
	Alcohol units per wk	3.02 (2.55)	3.55 (3.18)	2.99 (2.12)	2.60 (2.16)
**AUDIT^o^, n (%)**					
	Low-risk consumption	94.79 (7.19)	94.11 (8.69)	94.33 (5.50)	95.71 (6.86)
	Hazardous or risky consumption	4.93 (7.10)	5.88 (8.69)	4.71 (5.14)	4.28 (6.86)

^a^WC: waist circumference.

^b^HC: hip circumference.

^c^WHR: waist-to-hip ratio.

^d^SBP: systolic blood pressure.

^e^DBP: diastolic blood pressure.

^f^FPG: fasting plasma glucose.

^g^TG: triglyceride.

^h^LDL: low-density lipoprotein.

^i^HDL: high-density lipoprotein.

^j^HbA_1c_: glycated hemoglobin.

^k^HOMA: homeostatic model assessment.

^l^REGICOR-Framingham: Framingham-Registre Gironí del Cor

^m^MEDAS: Mediterranean Diet Adherence Screener.

^n^MET: metabolic equivalent of task.

^o^AUDIT: Alcohol Use Disorders Identification Test.

Patient-level baseline characteristics are summarized in Multimedia Appendix 7. The mean age of the patients was 59.79 (SD 9.75) years, and 54.5% (199/365) were female. Full details of the baseline characteristics have been reported elsewhere [36].

### Adherence to and Reach of the PREDIABETEXT Intervention

The intervention, consisting of SMS text message delivery, was implemented between February 21, 2022, and January 13, 2023. Over the 6-month intervention period, 246 individuals with prediabetes (n=106, 43.1% in intervention group A and n=140, 56.9% in intervention group B) received 72 intervention messages (3 per week) across 7 staggered cohorts initiated as participants were recruited. In addition, 2 reminder messages—one for the baseline visit and one for the final visit—were sent to each participant, bringing the total to 74 SMS text messages per participant.

A total of 18,204 SMS text messages were sent during the trial, of which 186 (1.02%) failed to be sent correctly. A total of 13.8% (34/246) of the participants missed at least one intervention message; however, no participant received <50% of the intended messages (<37 SMS text messages). Overall, 94.3% (232/246) of participants received at least 95% of the planned messages (≥70 SMS text messages). The messaging platform’s performance was monitored throughout the study to ensure consistent and reliable delivery.

### Primary Outcome

**Figure 2:**
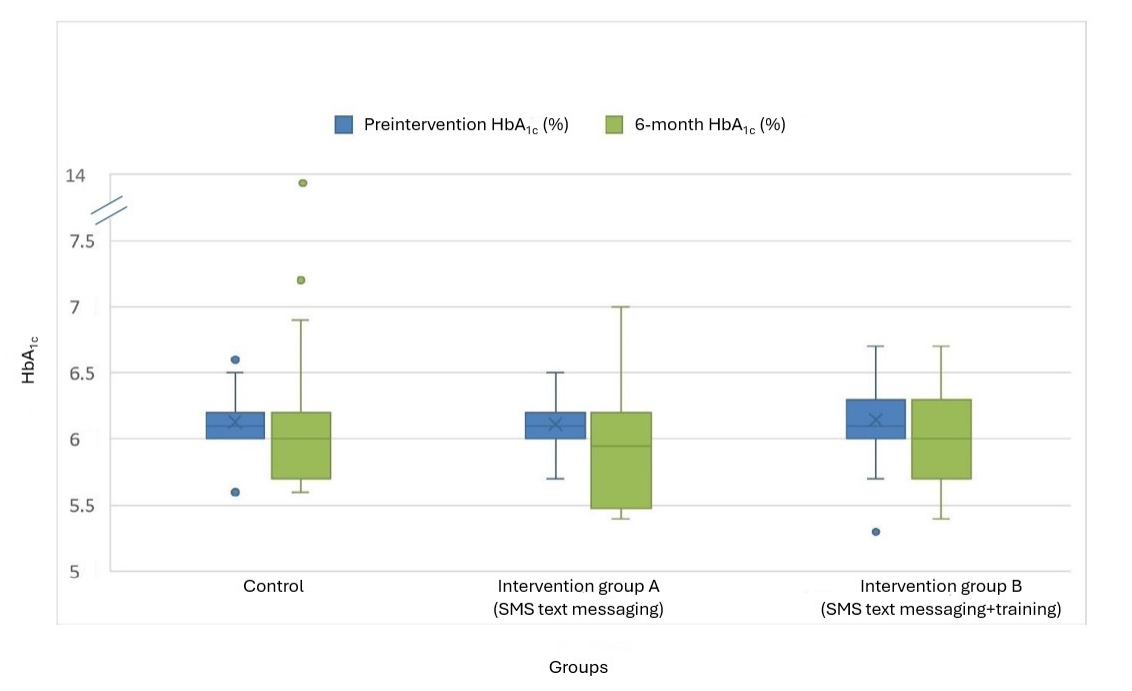
Glycated hemoglobin (HbA1c) levels across the 3 groups before the intervention and over the 6-month intervention period.

Raw data for the primary and secondary outcomes in intervention groups A and B and the control group at baseline and the 6-month time point are available in Multimedia Appendix 8. At 6 months, the mean HbA_1c_ level was 6.07% (SD 0.29%) in intervention group A, 6.12% (SD 0.25%) in intervention group B, and 6.18% (SD 0.79%) in the control group (Figure 2).

The ITT analysis showed no statistically or clinically significant differences in HbA_1c_ levels between the 2 intervention groups and the control group. Intervention group A was associated with a small, nonsignificant reduction in HbA_1c_ levels (β=−0.05, 95% CI −0.21 to 0.10; *P*=.50), whereas intervention group B also showed a nonsignificant reduction (β=−0.04, 95% CI −0.12 to 0.10; *P*=.56; Table 2).

**Table 2:** Effects of the digital intervention targeted at patients (intervention group A) and the combined digital intervention targeted at patients and health care professionals (intervention group B) on clinical, physiological, and behavioral outcomes (intention-to-treat analysis).

Variable			Intervention group A vs control				Intervention group B vs control		
			Imputed β (95% CI)		*P* value	Imputed β (95% CI)			*P* value
**Glycemic control**									
	HbA_1c_^a,b^	−0.05 (−0.21 to 0.10)		.50		−0.04 (−0.12 to 0.10)		.56	
	FPG^c,d^	−2.51 (−8.43 to 3.40)		.40		−2.25 (−7.77 to 3.25)		.41	
	Insulin^e^	1.24 (−3.37 to 5.86)		.59		−0.69 (−4.53 to 3.15)		.72	
	HOMA^f,g^	0.29 (−1.05 to 1.63)		.67		−0.41 (−1.66 to 0.82)		.50	
**Lipid profile**									
	Tg^b,h^	2.77 (−16.66 to 22.21)		.77		−5.99 (−20.72 to 8.73)		.42	
	Total cholesterol^b^	6.27 (−3.08 to 15.63)		.18		2.04 (−6.72 to 10.81)		.64	
	LDL^e,i^	10.33 (1.75 to 18.91)		.01		2.60 (−5.44 to 10.65)		.52	
	HDL^g,j^	−0.93 (−3.72 to 1.85)		.51		−0.44 (−2.96 to 2.07)		.72	
**Anthropometric outcomes**									
	Weight^b^	−0.48 (−1.86 to 0.90)		.49		−0.48 (−1.61 to 0.64)		.39	
	Waist circumference^d^	−0.64 (−2.30 to 1.01)		.44		−0.67 (−2.46 to 1.10)		.45	
	Hip circumference^e^	1.36 (0.87 to −0.39)		.12		0.22 (0.82 to −1.45)		.78	
	BMI^g^	−0.09 (−0.67 to 0.49)		.75		−0.14 (0.22 to −0.58)		.52	
**Cardiovascular outcomes**									
	REGICOR-Framingham^k^ score^b^	0.05 (−0.74 to 0.85)		.89		0.14 (−0.69 to 0.98)		.72	
	SBP^d,l^	−2.56 (−6.62 to 1.50)		.21		−0.47 (−4.43 to 3.47)		.81	
	DBP^e,m^	0.41 (−1.95 to 2.77)		.73		0.79 (−1.61 to 3.19)		.51	
**Lifestyle outcomes**									
	Adherence to Mediterranean diet (score of 0-14)^b^	0.49 (−0.06 to 1.04)		.08		0.25 (−0.27 to 0.79)		.34	
	Total energy expenditure in physical activity (MET^n^ min per wk)^d^	210.78 (−377.32 to 798.89)		.48		2.43 (−475.12 to 479.98)		.99	
	Sedentary lifestyle—NHS4Total^e,o^	0.25 (−0.29 to 0.79)		.37		0.36 (−0.12 to 0.86)		.14	
	Total alcohol units per wk^g^	0.95 (−0.53 to 2.45)		.20		0.86 (−0.50 to 2.23)		.21	

^a^HbA_1c_: glycated hemoglobin.

^b^Adjusted for baseline glycated hemoglobin.

^c^FPG: fasting plasma glucose.

^d^Adjusted for baseline fasting plasma glucose.

^e^Adjusted for baseline insulin.

^f^HOMA: homeostatic model assessment.

^g^Adjusted for baseline homeostatic model assessment.

^h^TG: triglyceride.

^i^LDL: low-density lipoprotein.

^j^HDL: high-density lipoprotein.

^k^REGICOR-Framingham: Framingham-Registre Gironí del Cor

^l^SBP: systolic blood pressure.

^m^DBP: diastolic blood pressure.

^n^MET: metabolic equivalent of task.

^o^NHS4Total: Nurses’ Health Study.

### Secondary Outcomes

#### Glycemic Control

The ITT analysis showed no significant differences in FPG between intervention group A (β=−2.51, 95% CI −8.43 to 3.40; *P*=.40) or intervention group B (β=−2.25, 95% CI −7.77 to 3.25; *P*=.41) and the control group. No significant differences were observed in insulin levels or homeostatic model assessment scores (Table 2).

#### Lipid Profile

A significant increase in LDL cholesterol was observed in intervention group A compared to the control group (β=10.33, 95% CI 1.75-18.91; *P*=.01). No significant differences were found in other lipid parameters, including triglycerides or HDL cholesterol, across the groups.

#### Anthropometric Outcomes

No significant differences were found in weight, waist circumference, or hip circumference between the groups in the ITT analysis. Although a minor difference in hip circumference was noted in the per-protocol analysis (Multimedia Appendix 9), it was not statistically significant under ITT analysis.

#### Cardiovascular Outcomes

No significant changes were observed in the REGICOR-Framingham cardiovascular risk score or in systolic and diastolic blood pressure across the groups in the ITT analysis.

#### Lifestyle Outcomes

The ITT analysis revealed no significant differences between groups in adherence to the Mediterranean diet, total energy expenditure in physical activity (measured in MET minutes per week), sedentary behavior, or alcohol consumption.

### Risk of Progression to Diabetes

The risk of progression to diabetes is shown in Multimedia Appendix 10. The GEE model for the risk of progression to diabetes showed a lower risk of progression to diabetes for both intervention groups A (adjusted odds ratio 0.51, 95% CI 0.12-2.21) and B (adjusted odds ratio 0.49, 95% CI 0.12-1.94) versus the control group, but this effect was not statistically significant.

### Impact on Health Care Professional Knowledge About Prediabetes and Communication Skills

Among the 18 health care professionals in intervention group B who completed the online training cointervention, the knowledge score immediately after completing the course was significantly higher (mean 12.17, SD 2.20) compared to baseline (mean 6.83, SD 1.33). However, after the 6 months of the intervention, the knowledge score in intervention group B (mean 8.89, SD 3.92) did not significantly differ from that in intervention group A (mean 6.33, SD 2.06) or the control group (mean 7.09, SD 1.37), neither of which received the educational intervention (full details are available in Multimedia Appendix 11).

### Embedded Postintervention Qualitative Interviews

#### Overview

A total of 23 patients were invited to participate in the interviews, selected for diversity in age, gender, and nationality, of whom 8 (35%) agreed to participate. Similarly, 20 health care professionals were invited, and 7 (35%) agreed to participate. The main reasons for nonparticipation included a lack of response to phone calls and being unavailable at the proposed time. The qualitative analysis identified key themes regarding the perceived benefits, challenges, and areas for improvement of the PREDIABETEXT intervention among both patients and health care professionals.

#### Patient Perspectives

##### Perceived Benefits

Patients reported high satisfaction with the intervention, particularly the dietary messages, which were especially well received by younger participants. The message frequency was considered appropriate, and many participants expressed interest in continuing to receive them:

[The diet SMS text messages] were quite interesting because at the end, I knew a little bit about the quantities or the topic of vegetables or how to organize a dish, and they were quite good.

Woman; aged 25 years

Well, for me it had been very, very interesting. And it has helped me a lot to control day by day what I consumed and everything I had to do. In other words, I followed them a lot. In fact, I managed to lose a lot of weight [...] The messages are very positive; they have helped me a lot.

Man; aged 51 years

I’m fine with the frequency, because if we start bombarding [messages], you get bored. But the timing of when they’re sent is working very well for me...

Man; aged 37 years

##### Suggested Improvements

Patients recommended greater personalization, links to recipe websites, and periodic in-person meetings or nursing follow-ups at 3 months:

I don’t know, maybe meet once a month, but in person or something like that...

Woman; aged 72 years

Maybe it’s an idea...to quote them so that people can evaluate it again...every three months or four months...

Man; aged 37 years

##### Behavioral Impact

Some participants reported dietary improvements and increased physical activity, although sustained weight loss was only mentioned by 12% (1/8) of the patients:

Good, because that way, look, you get used to leading a healthy life...In the mornings, I do 1 hour of yoga and another of Pilates, and then I walk...And then in the afternoon, I also do about 2 hours of walking...Sometimes 18,000, sometimes 17,000, but sometimes, yes, 20,000, yes.

Man; aged 37 years

Also automatically, as I have taken more care of myself with food, I have cooked healthier for the whole family.

Woman; aged 50 years

##### Actionable Recommendations

Future iterations of the intervention could incorporate personalized content, integrate external resources (eg, recipe links), and explore hybrid models that combine digital support with periodic face-to-face interactions.

#### Health Care Professional Perspectives

##### Perceived Benefits

The online training was deemed valuable in increasing awareness of prediabetes management:

In my opinion, it has helped me a little to keep it in mind, I mean, to think that they do exist, that they have always existed, but it’s like we are so focused on treating diabetes, treating hypertension that we don’t think about prevention.

Physician; woman; aged 35-50 years

...it has inspired us to take this topic to the street, to the patients, to the people who we have not yet done so. The user patients.

Nurse; woman; aged 50-65 years

##### Challenges

Time constraints were a key barrier to participation, and preferences varied regarding the training format (in person, online, or blended):

When it is face-to-face, you can interact with the speaker. Here, if a question comes up, you can put it in the chat. It is not the same as a reply, counter-reply, or clarification of doubts, because when you are writing, maybe you do not express yourself well or the other party does not understand what you wanted to say. You can manage all this better in person.

Physician; man; aged 50-65 years

##### Suggested Improvements

Participants recommended a shorter course duration and highlighted the importance of ensuring that nursing staff also receive the training:

In an overcrowded office, unless you have the cooperation of your nurse, you can’t do a proper intervention. That is clear.

Physician; woman; aged 35-50 years

I think it’s well designed for two or four [weeks], because things can always come up that prevent you from doing it in the time you initially planned. So, at first glance, it seems good to me.

Physician; man; aged 50-65 years

##### Actionable Recommendations

To improve uptake, the training could be streamlined, offered in a blended format, and specifically targeted to multidisciplinary primary care teams.

## Discussion

### Principal Findings

This 6-month pragmatic RCT conducted within primary health care settings as part of routine practice evaluated a multifaceted digital lifestyle intervention grounded in multiple behavior change theories. The intervention did not significantly reduce HbA_1c_ levels, the primary outcome, when compared to the control group receiving usual care.

The PREDIABETEXT intervention, carefully co-designed with end users, offers several strengths. Clinically, its scalability at a low cost makes it a sustainable option for health care institutions. To address the low participation and retention rates often observed in high-intensity, in-person programs such as the Diabetes Prevention Program, we opted for a low-intensity intervention. In addition, delivering PREDIABETEXT within a real-world setting leveraging the resources of the Balearic Islands health service (eg, secure access to electronic health records, SMS text message communication, and the Moodle platform for online training) provided 2 key benefits: a more accurate assessment of the intervention’s impact in routine clinical practice and the potential for its immediate integration into the health system if proven effective. This approach is in line with that of similar studies reporting that the use of remotely administered interventions was convenient for participants with prediabetes [37].

The intervention did not demonstrate a statistically significant improvement in glycemic control. Although there was a reduction in HbA_1c_ levels and FPG values for intervention groups A and B compared to the control group, this effect was not statistically significant. Previous literature in this area presents heterogeneous results. A recent systematic review evaluating the efficacy of remotely administered lifestyle interventions for preventing T2DM found that 6 out of 8 interventions resulted in significant reductions in weight or glycemic biomarkers such as HbA_1c_ and fasting glucose [37]. A long-term study of an internet-based diabetes prevention program demonstrated a reduction in HbA_1c_ levels by a mean of 0.38% (SD 0.07%) after 1 year and 0.43% (SD 0.08%) after 2 years [38], supporting the notion that both multimodal and long-term interventions can yield meaningful results. Furthermore, a systematic review and meta-analysis showed that digital health interventions resulted in a 12% risk reduction in T2DM incidence (risk ratio=0.88, 95% CI 0.77-1.01; *I*^2^=0.6%; *P*=.06) compared to the control group [39]. Consistent with our findings, a systematic review reported a controversial effect of digital health interventions on HbA_1c_ and FPG values [14]. In addition, a study in Spain evaluating the effects of a low-intensity nurse-led lifestyle intervention on glycemic control in individuals with prediabetes did not find statistically significant differences in FPG at 9 months [40]. Several factors may explain the results of our intervention. First, it was low intensity and relatively short in duration (6 months), whereas previous studies suggest that more comprehensive, long-term interventions may yield greater reductions in diabetes risk. Second, participants had relatively mild metabolic alterations at baseline, and adherence to behavior change recommendations may have varied, potentially affecting outcomes. Finally, while the study was sufficiently powered to detect changes in HbA_1c_ levels, it may have been underpowered to detect small but meaningful reductions in diabetes incidence.

The findings of our study showed no significant beneficial effect on triglyceride, cholesterol, HDL, or LDL levels. These results align with those of a systematic review and meta-analysis that reported minimal effects on lipid parameters in participants with prediabetes using digital health interventions [14].

Regarding the anthropometric measures of study participants, similar to our study, a long-term study of an internet-based diabetes prevention program showed no significant effect on body weight after 1 or 2 years of follow-up [38]. In contrast, the findings of a systematic review by Jeem et al [14] showed a positive effect of mHealth interventions on anthropometric measures such as body weight, waist circumference, and BMI among middle-aged and older patients with prediabetes. A meta-analysis estimated the overall effect of stand-alone eHealth interventions on T2DM prevention to be a mean weight loss of −3.34% (95% CI −4% to −2.68%) from baseline to up to 15 months of follow-up across all interventions [41]. Another meta-analysis that evaluated the effect of technology-mediated diabetes prevention interventions on weight showed a pooled weight loss effect of 3.76 kg (95% CI 2.8-4.7; *P*<.001), as well as improvements in glycemia in patients with prediabetes [42].

Regarding cardiovascular outcomes, the ITT analysis did not reveal any significant differences between the groups in systolic and diastolic blood pressure or the REGICOR-Framingham score. Previous systematic reviews and meta-analyses have focused primarily on measuring blood pressure and have not assessed cardiovascular risk scores. In this regard, our trial provides new data. The REGICOR-Framingham score is a highly relevant outcome measure for evaluating the success of interventions aimed at preventing T2DM due to its strong association with cardiovascular disease, a major complication of T2DM. Patients with T2DM are at significantly elevated risk of cardiovascular disease, with relative risks approximately 1.8 to 3.3 times higher than those for individuals without diabetes [43]. According to a study a reduction of 1% to 2% in the 10-year cardiovascular risk is generally considered clinically significant [44]. Thus, accurately assessing and reducing cardiovascular risk, which can be achieved through the REGICOR-Framingham score [45], not only supports T2DM prevention efforts but also addresses the leading cause of morbidity and mortality in this population. Future research should explore higher-intensity, personalized digital health interventions; extend the intervention period; and test strategies to enhance patient engagement and adherence.

### Strengths and Limitations

One of the strengths of our study is the use of validated questionnaires, which enhanced both the internal and external validity of our findings. The use of blinded outcome evaluators strengthened internal validity, whereas the representativeness of the recruited patients, reflecting the general population with prediabetes of the Balearic Islands, improved external validity.

However, this trial has several limitations. Information and recall biases may have been introduced by measuring physical activity and eating habits through self-reported data. To mitigate these biases, objective measures such as pedometers, 24-hour dietary recalls, or food frequency questionnaires could have been used to provide a more accurate assessment.

Another potential limitation is the choice of assessment tool for cardiovascular risk. While measures such as the American College of Cardiology/American Heart Association pooled cohort equation may provide broader insights, especially in diverse populations, we selected the REGICOR-Framingham score due to its strong association with cardiovascular disease. This equation has been specifically adapted and validated for the Spanish population, making it particularly suitable for evaluating intervention effectiveness in our setting. Future research should consider incorporating more contemporary and widely applicable risk assessment tools to enhance generalizability across different populations.

Although multiple secondary outcome comparisons were conducted without formal statistical adjustment, most did not show significant changes. Nevertheless, the findings should be interpreted with caution due to the increased risk of type I error.

The Hawthorne effect may have influenced the results, potentially leading to an underestimation of the intervention’s effect. Participants in the control group may have altered their behavior simply because they were aware of being part of a study, thereby narrowing the differences between the intervention and control groups.

Moreover, the generalizability of the study may be limited by the exclusion of non–Spanish-speaking individuals and minority groups. In addition, hard-to-reach populations were likely underrepresented as one key eligibility criterion required participants to have a recent test result. Socially vulnerable individuals, who are more likely to develop T2DM but less likely to visit their primary care physician, may not have been adequately represented in this study. Future research should explore and implement recruitment strategies that more effectively engage these underserved groups. This study was conducted in Mallorca, Spain, which might limit the generalizability of the results to other populations or settings. Furthermore, the duration of the study was 6 months, which might be too short to capture the long-term effects of the intervention. Evaluating HbA_1c_ levels at 6 months offers useful short-term insights; however, extending the intervention period in future research could provide a more comprehensive understanding and help mitigate the effects of regression to the mean, ensuring more reliable long-term conclusions.

Another limitation at the health care professional level was that our 16-hour training program might be too lengthy, potentially leading to time constraints in applying the training. Moreover, to enhance knowledge retention, future studies should consider incorporating reinforcement sessions at 1- to 3-month intervals as strategic reminders. In addition, we prioritized health care professionals with a higher number of patients meeting the prediabetes criteria for inclusion. Although this strategy might introduce selection bias, it is unlikely to have affected our findings.

Finally, the cost-effectiveness of the PREDIABETEXT intervention was not assessed in this study. Future research should include a comprehensive cost-effectiveness analysis to evaluate the scalability and sustainability of the intervention.

### Conclusions

In conclusion, this multifaceted digital intervention for the prevention of T2DM in the primary care setting did not significantly improve glycemic control in individuals with prediabetes who received SMS text messages or those whose health care professionals also received training compared to the usual care control group. On the basis of these findings, future research should prioritize the development and evaluation of higher-intensity digital health interventions that include more frequent interactions and personalized assignments to enhance participant engagement. Wearable devices offer real-time monitoring and personalized feedback, which can improve patient self-management and intervention outcomes. These interventions may be more effective when combined with intensive, multimodal approaches delivered simultaneously to at-risk populations. In addition, expanding research across multiple countries and focusing on the recruitment of underserved groups will improve the generalizability of the findings. Conducting real-world evidence studies to evaluate the effectiveness of digital health interventions in diverse populations and settings using electronic health records and other real-world data sources to assess outcomes is also recommended.

Furthermore, applying implementation science frameworks to study the integration of digital health interventions into routine clinical practice is recommended. This approach will help identify facilitators of and barriers to implementation and develop strategies to enhance the adoption and scalability of these interventions.

By addressing these priority research questions and using the recommended methodologies, future studies can advance the field of digital health interventions for diabetes prevention, ultimately improving health outcomes and informing health care policy and practice.

## Appendix

Multimedia Appendix 1 CONSORT-EHEALTH checklist (V 1.6.1).

Multimedia Appendix 2 Template for Intervention Description and Replication checklist—PREDIABETEXT (Prediabetes Text Message Digital Intervention for the Prevention of Type 2 Diabetes Mellitus) intervention.

Multimedia Appendix 3 Examples of SMS text messages delivered during the PREDIABETEXT (Prediabetes Text Message Digital Intervention for the Prevention of Type 2 Diabetes Mellitus) intervention.

Multimedia Appendix 4 Recruitment and randomization processes of the study participants.

Multimedia Appendix 5 Questionnaire assessing knowledge of prediabetes management among health care professionals.

Multimedia Appendix 6 Interview guides used in the within-trial process evaluation qualitative study.

Multimedia Appendix 7 Baseline demographic and clinical characteristics of the patients by the 3 trial arms.

Multimedia Appendix 8 Clinical characteristics of intervention group A, intervention group B, and the control group at baseline and the 6-month time point.

Multimedia Appendix 9 Effects of the digital intervention targeted at patients (intervention group A) and the combined digital intervention targeted at patients and health care professionals (intervention group B) on clinical, physiological, and behavioral outcomes (per-protocol analysis).

Multimedia Appendix 10 Risk of progression to type 2 diabetes at the 6-month time point.

Multimedia Appendix 11 Health care professionals’ qualification scores (0-15) before and after the training program and at the 6-month time point. .

## Abbreviations

CONSORT: Consolidated Standards of Reporting Trials
FPG: fasting plasma glucose
GEE: generalized estimating equations
HbA1c: glycated hemoglobin
HDL: high-density lipoprotein
ITT: intention-to-treat
LDL: low-density lipoprotein
MET: metabolic equivalent of task
mHealth: mobile health
PREDIABETEXT: Prediabetes Text Message Digital Intervention for the Prevention of Type 2 Diabetes Mellitus
RCT: randomized controlled trial
REGICOR: Framingham-REgistre GIroní del COR
T2DM: type 2 diabetes mellitus

## Data Availability

The datasets generated or analyzed during this study are available from the corresponding author on reasonable request.
