# Supplementary material for: A Multifaceted Digital Intervention for the Prevention of Type 2 Diabetes Mellitus in Primary Care (PREDIABETEXT): Cluster Randomized Trial
Source: J Med Internet Res. 2025 Oct 9;27:e70981. doi: 10.2196/70981 (PMC12550449; doi:10.2196/70981)
Supplement: Multimedia Appendix 2 [file jmir_v27i1e70981_app2.pdf]

TIDieR

# PREDIABETEXT (PREDIABEtes TEXT message digital intervention for the prevention of type 2 diabetes mellitus)

**Why:**

To promote healthy lifestyles and adherence to lifestyle recommendations to improve glucose tolerance, promote weight loss, and lower glycated hemoglobin (HbA1c) levels in people with prediabetes.

**What (material):**

The PREDIABETEXT intervention consisted of 3 personalized short-text messages per week to support lifestyle behavior changes. We started from the DiabeText SMS library (10.1016/j.ijmedinf.2023.105103) from which the messages were adapted for the pre-diabetes population based on last available guidelines from the American Diabetes Academia (doi:https://doi.org/10.2337/dc25-S003). The DiabeText library was created from an expert series of workshops with endocrinologists, nutritionists, sports scientists, nurses, psychologists, GPs and pharmacists, which generated up to 1000 messages using different behaviour change techniques (https://doi.org/10.1016/j.cct.2023.107399). Afterwards, a team of nutritionists and sports scientists picked and adapted the messages from DiabeText and created new messages with the aim of having messages covering all the relevant topics (motivation, type of food, portions, physical activity, sedentary lifestyle, etc.) adapted for the pre-diabetes population. More details of the intervention development are available at:
DOI: 10.3390/ijerph192214706

**What (procedures):**

The PreDiabeText Technological System for the messaging intervention. Three databases (clinical databases, database of brief messages, and patient-reported data) were combined using specific algorithms to produce a text file containing the information, which was uploaded to the SMS delivery platform and sent personalized SMSs to the study participants.

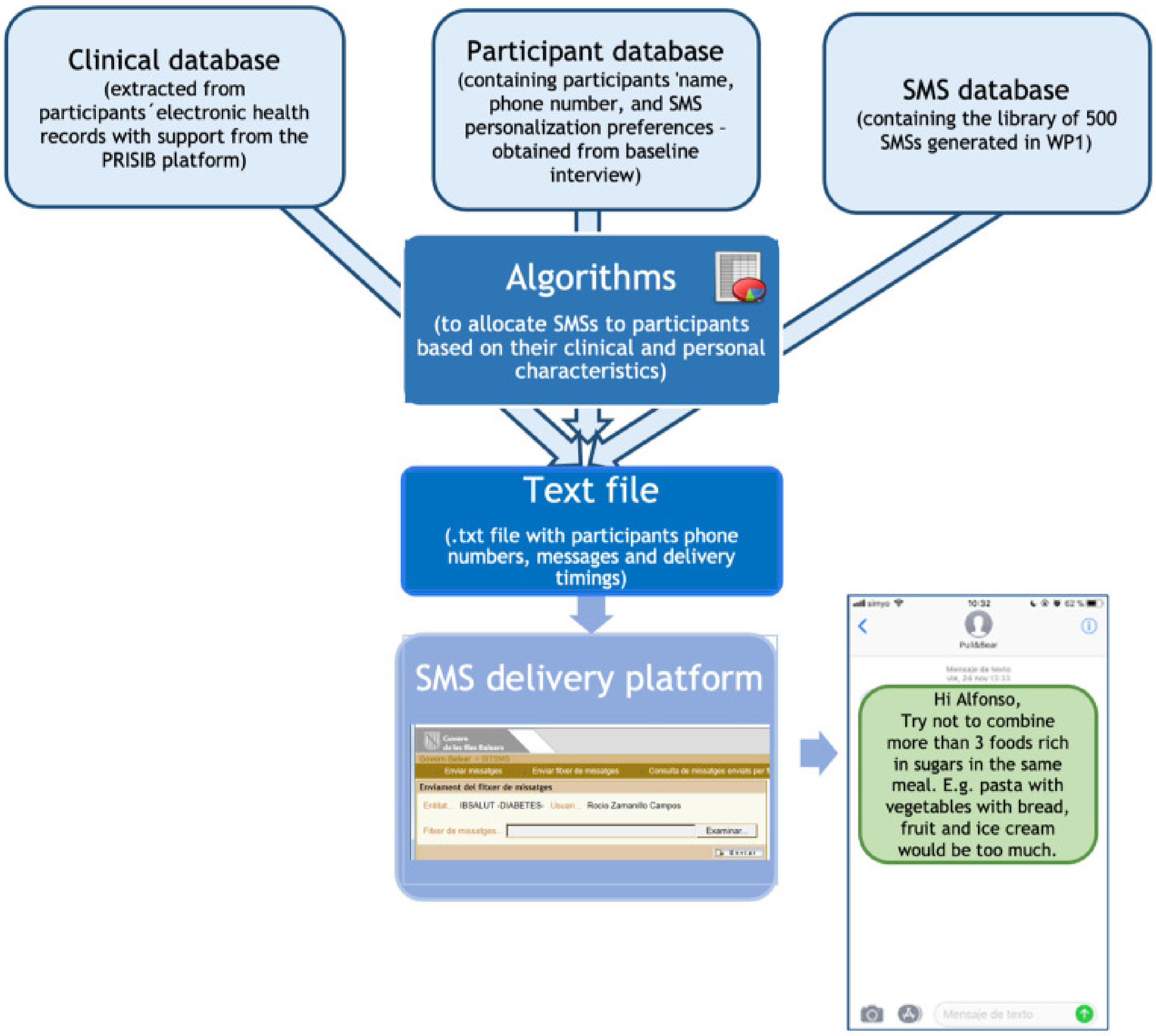

| | |
|---|---|
| **Who provided:** | The PREDIABETEXT intervention was provided by the project staff through the unique technological communication (SMS) service available for the public health services of the Balearic Islands which is called Bitmessage and was previously created and integrated by Fundacio Bit (https://www.fundaciobit.org/es/area-de-desarrollo/) for IbSalut (https://www.ibsalut.es/es/). |
| **How (mode of delivery; individual or group):** | Individual short text messages (160 characters maximum) were sent to the mobile phones of participants. |
| **Where:** | The PREDIABETEXT is a mobile health intervention without a specific location to be held. However, participants should remain in Spain during the study because the technological system is not able to send SMSs out of national borders without extra costs. The communication channel is unidirectional and asynchronous. |
| **When and how much:** | A total of 72 SMS messages (at a frequency of 3 messages per week) were sent over 6 months. Messages were queued early in the morning and reached participants at different times of the day, depending on the demands of the communication service. |
| **Tailoring:** | Messages were tailored based on participants' body weight, smoking status, and mobile Internet use. |
| **Modification:** | No modifications were introduced in the PREDIABETEXT intervention during the study. |
| **How well (planned):** | Research staff checked that all participants were receiving the intervention correctly by using the Bitmessage platform (which keeps a record of the SMS successfully delivered). We also programmed the intervention to send messages to two phone numbers from the research team with anonymous data from two participants in the control group, to check that SMSs were correctly sent and received. At the end of the follow-up, we asked all participants in the intervention group if they received the SMSs properly. |
| **How well (actual):** | According to the meta-data extracted from the Bitmessage message platform, a total of 186 messages were identified that were not sent correctly, which represents 1.02% of the total messages sent.<br>34 patients stopped receiving at least 1 of the intervention messages. However, of the total number of participants, no one received less than 50% of the intervention (<37 SMS).<br>94% of participants (n = 232) received at least 95% of the intervention (n = 70 SMS). |
