# Supplementary material for: A Multifaceted Digital Intervention for the Prevention of Type 2 Diabetes Mellitus in Primary Care (PREDIABETEXT): Cluster Randomized Trial
Source: J Med Internet Res. 2025 Oct 9;27:e70981. doi: 10.2196/70981 (PMC12550449; doi:10.2196/70981)
Supplement: Multimedia Appendix 3 [file jmir_v27i1e70981_app3.docx]

Online Appendix 3.  Examples of short text messages delivered by the PREDIABETEXT intervention.

| **Topic** | **Some examples of messages** |
| --- | --- |
| Diet | It is recommended that only a quarter of your plate be carbohydrates. Remember which vegetables are rich in carbohydrates (potato, sweet potato, pumpkin, corn).  It is important to stay hydrated throughout the day. If you exercise, drink water before, during, and after exercise. |
| Exercise | Don't stand for more than 60 minutes. Move around (walk, do chores, etc.) to benefit blood sugar control.  Doing more than 150 minutes of physical exercise per week reduces the risk of diabetes, heart disease, hypertension and many others. |
| Motivation | Are you overwhelmed and need to relax? Here we offer you several audios to relax with mindfulness or guided meditation: <https://cutt.ly/Jtz9xPB>  Set short-term goals, it will be easier for you to achieve your goals! |
| General information about prediabetes | Prediabetes indicates alterations in blood sugar levels, but you are in time to reverse them. Don't let it turn into diabetes!  Prediabetes is a condition in which blood sugar levels are higher than normal: fasting glucose 100-125 mg/dL or glycosylated hemoglobin 5.7-6.4% |
| Smoking cessation | Smoking is even more harmful to people with high blood sugar levels. Quitting is easier with help. Ask your health centre for information. |
