# Supplementary material for: A Multifaceted Digital Intervention for the Prevention of Type 2 Diabetes Mellitus in Primary Care (PREDIABETEXT): Cluster Randomized Trial
Source: J Med Internet Res. 2025 Oct 9;27:e70981. doi: 10.2196/70981 (PMC12550449; doi:10.2196/70981)
Supplement: Multimedia Appendix 4 [file jmir_v27i1e70981_app4.docx]

Appendix 4. Recruitment and Randomization Processes.

| Category | Healthcare Professionals | Patients |
| --- | --- | --- |
| Eligibility Criteria | Age: 18-75 years Registered with the Public Health Service of the Balearic Islands Prioritized based on the number of patients meeting prediabetes criteria Exclusions: Planning to transfer to another primary healthcare unit | Age: 18-75 years Registered with the Public Health Service of the Balearic Islands Prediabetes criteria: HbA1c 6-6.4%, FPG 110-125 mg/dL, or both Access to a mobile device capable of receiving SMS Exclusions: Unable to read messages in Spanish, severe mental health conditions, currently on antidiabetic medications, recent childbirth or pregnancy, planning to change healthcare centers |
| Recruitment Process | Invited via email Confirmed via phone calls | Identified from primary care databases Invited via SMS Confirmed via phone calls |
| Randomization Process | Randomized into three groups using computer-generated random numbers | Assigned to intervention groups based on their healthcare professional's allocation |
| Intervention Details | Intervention A: Patient SMS 3 SMS/week Topics: Nutrition, Physical Activity, Motivation Intervention B: Patient SMS + Provider Training 3 SMS/week (same as Intervention A) 16-hour online training for healthcare professionals Topics: Prediabetes management, behavior change, clinical guidelines Control Group: Usual Care Standard primary care services | Intervention A: Patient SMS 3 SMS/week Topics: Nutrition, Physical Activity, Motivation Intervention B: Patient SMS + Provider Training 3 SMS/week (same as Intervention A) 16-hour online training for healthcare professionals Topics: Prediabetes management, behavior change, clinical guidelines Control Group: Usual Care Standard primary care services |
