# Supplementary material for: A Multifaceted Digital Intervention for the Prevention of Type 2 Diabetes Mellitus in Primary Care (PREDIABETEXT): Cluster Randomized Trial
Source: J Med Internet Res. 2025 Oct 9;27:e70981. doi: 10.2196/70981 (PMC12550449; doi:10.2196/70981)
Supplement: Multimedia Appendix 5 [file jmir_v27i1e70981_app5.docx]

Multimedia Appendix 5. Questionnaire assessing knowledge of prediabetes management among health care professionals.

1. **A 43-year-old male patient of Asian descent with a Body Mass Index (BMI) of 27 who is apparently healthy presented with back pain. His medical history revealed no significant illnesses, and he had not undergone laboratory tests in the last 5 years. What is the likelihood that this patient has prediabetes?**
2. Between 2 and 5%
3. Between 5 and 10%
4. Between 25 and 45%
5. Between 50 and 65%

**Correct answer: c**

Justification: Prediabetes is a prevalent and often underdiagnosed public health issue. Its prevalence (27% to 49%, depending on the diagnostic criteria) varies by age, sex, ethnicity, region, and socioeconomic status. In Spain, approximately 14.8% of the population has prediabetes. A study of the working population (20–65 years old) in the Balearic Islands reported a prevalence of 12%. In 2020, the CDC in the United States estimated that 34.5% of adults had prediabetes. However, only 15.4% of individuals with prediabetes are diagnosed, as it typically presents with no symptoms.

**2. If a blood test on the above patient reveals a fasting plasma glucose level of 114 mg/dL and no intervention is performed, what is the probability of progression to type 2 diabetes within 5 years?**

1. Approximately 5%
2. Between 5 and 10%
3. Between 10 and 15%
4. Between 25 and 50%

**Correct answer: d**

Justification: The annual progression rate from prediabetes to type 2 diabetes is 5–10%, with an estimated 70% of individuals with prediabetes developing diabetes during their lifetime. Studies have shown that over 3–5 years, approximately 25% of individuals progress to type 2 diabetes, while 50% remain prediabetic.

**3. If a diet and exercise intervention is performed for the previous patient, by how much is the risk of developing type 2 diabetes reduced?**

1. Does not reduce
2. Is reduced by 20%
3. Can be reduced by half
4. Disappears

**Correct answer: c**

Justification: Two major clinical trials, the Diabetes Prevention Program (DPP) in the United States and the Finnish Diabetes Prevention Study (DPS), showed that lifestyle interventions can delay or prevent type 2 diabetes in individuals with prediabetes, reducing the risk by approximately 50%. The DPP demonstrated that lifestyle interventions were more effective than metformin (58% vs. 38% risk reduction). The greatest risk reduction occurred in individuals who lost weight through diet and exercise, with benefits sustained at 10 years of follow-up.

**4. A balanced plate for people with prediabetes should:**

1. Be the same as the balanced plate for the general population.
2. Include fruit in the quarter of the plate for carbohydrate-rich foods.
3. Have an asterisk in the middle of the vegetable section warning people to avoid starchy vegetables.
4. Promote plant-based proteins in a quarter of the plate for protein-rich foods.

**Correct answer: b**

Justification: For prediabetes, a balanced plate includes fruit in the carbohydrate-rich quarter. Clinical evidence supports the reduction of total carbohydrate intake in individuals with diabetes and prediabetes. The American Diabetes Association recommends similar dietary guidelines for prediabetes and diabetes, emphasizing moderate carbohydrate intake, including fruit, in a balanced diet.

**5. A legume stew is a balanced dish if it meets the following criteria:**

1. Meat should cover one-quarter of the plate for protein-rich foods.
2. Potatoes are added to cover one quarter of the plate with carbohydrate-rich foods.
3. Incorporate vegetables and greens to cover half the plate with foods rich in minerals and vitamins.
4. Add fruit to the same pot.

**Correct answer: c**

Justification: A balanced legume stew should include half the plate filled with non-starchy vegetables rich in minerals and vitamins. Legumes provide carbohydrates and proteins, making additional protein- or carbohydrate-rich foods (e.g., meat, potatoes, tuna, cheese, chorizo, ham, crab sticks, chicken, fried plantains, and corn) unnecessary. This approach promotes meal balance and reduces caloric intake. Legumes can be served hot in winter or as a salad in summer; however, including sufficient non-starchy vegetables is key in both cases.

**6. In nutritional assessment using the fourteen-point PREDIMED questionnaire, identify the false or incorrect statement:**

- 1. Wine consumption was excluded, and the results were evaluated using 13 points.
  2. Fish servings per week included canned, smoked, frozen fish, and shellfish.
  3. A fruit serving is equivalent to the size of a person’s fist.
  4. Two weekly legume servings were sufficient to earn 1 point for this item.

**Correct answer: d**

Justification: The PREDIMED questionnaire awards 1 point for consuming three or more servings of legumes per week, not two servings as stated in option d.

**7. In individuals with prediabetes who are overweight or obese:**

1. Begin weight loss intervention immediately.
2. Evaluate and qualitatively improve the dietary intake.
3. First, identify the type of eating behavior (balanced, continuous snacking, or compulsive eating).
4. First, evaluate the predisposition to change habits.

**Correct answer: c**

Justification: Understanding eating habits is crucial as they guide the recommendations and strategies used during consultations. For example, if recurring issues hinder a balanced diet, purely dietary strategies should be avoided. Instead, address motivational and problem-solving aspects of behaviors like continuous snacking or compulsive eating.

**8. In which patients recently diagnosed with prediabetes would you consider adding drug therapy in addition to lifestyle modifications based on diet and exercise?**

1. Everyone systematically.
2. Those who prefer it over lifestyle changes.
3. Especially those with BMI > 35 kg/m².
4. Individuals over 65 years of age, given the higher incidence of diabetes with age.

**Correct answer: c**

Justification: The incidence of prediabetes and its progression to type 2 diabetes mellitus is higher in obese individuals, particularly those with a BMI > 35 kg/m². Clinical trials have supported the use of metformin in these patients. Drug therapy should be individualized based on patient-specific factors and not applied systematically. Non-pharmacological interventions, such as lifestyle changes with diet and exercise, should always be the first approach to treatment. Older adults (>65 years) have a lower progression rate from prediabetes to diabetes and often experience polypharmacy and frailty, making drug therapy a less suitable option.

**9. In a patient with obesity and prediabetes, which therapeutic strategy would you NOT consider for managing prediabetes?**

1. Metformin
2. Dipeptidyl peptidase 4 (DPP-4) inhibitors
3. Pioglitazone
4. Liraglutide

**Correct answer: b**

Justification: Metformin is the first-line pharmacological option for prediabetes, supported by studies demonstrating its efficacy, safety, and cost-effectiveness. DPP-4 inhibitors lack robust evidence for preventing progression to type 2 diabetes in prediabetes and have a neutral effect on weight, making them less suitable. In the ACT NOW study, pioglitazone (45 mg) reduced the progression to type 2 diabetes by 72%, with lower doses improving insulin sensitivity while causing less weight gain and fluid retention; however, its adverse effects make its use controversial. The SCALE trial showed that liraglutide (3 mg) promotes sustained weight loss and reduces the progression to type 2 diabetes in obese patients with prediabetes. Although more expensive and not always funded for this indication, liraglutide is a viable option for selected patients.

**10. Regarding vascular complications in prediabetes, identify the false statement:**

1. Prediabetes is a cardiometabolic state associated with an increased risk of macrovascular and microvascular complications.
2. Patients with impaired fasting glucose (IFG) have a higher cardiovascular risk than those with impaired glucose tolerance (IGT).
3. Screening for type 2 diabetes or prediabetes is recommended for patients with new-onset ischemic heart disease.
4. Cardiovascular risk factors (dyslipidemia, obesity, and high blood pressure) are highly prevalent in individuals with prediabetes.

**Correct answer: b**

Justification: Studies show that impaired glucose tolerance (IGT) is a stronger predictor of macrovascular complications, such as cardiovascular disease, than impaired fasting glucose (IFG). Therefore, the statement that IFG carries a higher cardiovascular risk than IGT is incorrect. Prediabetes is associated with an increased risk of macro- and microvascular complications (option a), screening is recommended for new-onset ischemic heart disease (option c), and cardiovascular risk factors are highly prevalent in prediabetes (option d), all of which are true.

**11. When following up with patients with prediabetes, we should:**

1. Treat cardiovascular risk factors (CVRFs) with the same therapeutic objectives as patients in secondary prevention.
2. Treat CVRFs with the same therapeutic goals as patients with type 2 diabetes.
3. Treat CVRFs with the same therapeutic goals as the general population, calculating their overall cardiovascular risk and adapting goals accordingly.
4. Treat CVRFs with the same therapeutic goals as low cardiovascular risk patients.

**Correct answer: c**

Justification: Cardiovascular risk factors (CVRFs), including dyslipidemia, obesity, and hypertension, are highly prevalent in prediabetes. It is unclear whether the higher cardiovascular risk in prediabetes is due to prediabetes itself or is associated with CVRFs. Therefore, CVRF treatment goals for prediabetes align with those for the general population based on the calculated overall cardiovascular risk, with closer monitoring to identify and manage these risk factors.

**12. What are the physical activity recommendations for adults with prediabetes?**

1. Combine light physical activity with stretching.
2. Combine 150–300 minutes of moderate-to-vigorous physical activity per week with resistance training, two or more days a week.
3. Walk for 10–15 minutes every day.
4. Engage in 60 minutes of physical activity daily, choosing between aerobic, HIIT, balance, or flexibility exercises.

**Correct answer: b**

Justification: Adults with prediabetes should follow the physical activity recommendations for their age group. According to the WHO 2020 guidelines, adults aged 18–64 years should perform 150–300 minutes of moderate-to-vigorous physical activity weekly, plus resistance training on two or more days per week. This approach improves insulin sensitivity and reduces the risk of progression to type 2 diabetes mellitus.

**13. How often is physical activity recommended for individuals with prediabetes?**

1. Practice physical activity regularly, with no more than two days between sessions.
2. Frequency is not relevant as long as the weekly recommendations are met.
3. Daily physical activity is required to control blood sugar levels.
4. Practice physical activity regularly, with no more than four days between sessions.

**Correct answer: a**

Justification: The benefits of exercise on glucose levels last for up to 72 hours. Thus, physical activity should occur at least every 48 hours to sustain glycemic control in prediabetes, ensuring regular exercise with no more than two days between sessions.

**14. Which of the following statements is correct regarding physical activity in the management of prediabetes?**

1. Resistance exercises are key to controlling prediabetes by improving glucose metabolism, as greater muscle mass enhances insulin uptake.
2. Aerobic exercise is the most important factor in managing prediabetes, regardless of resistance training.
3. Individualization is the key. Combining resistance, aerobic, and balance exercises or focusing on one type may be more beneficial depending on the individual.
4. Both A and B are correct.

**Correct answer: a**

Justification: Resistance training increases muscle mass, improving glucose metabolism and insulin uptake, making it especially important for controlling prediabetes. According to the WHO 2020 guidelines, adults aged 18–64 years should combine 150–300 minutes of moderate-to-vigorous aerobic exercise weekly with resistance training on two or more days per week, with balance exercises added for those over 65 years. Aerobic and resistance exercises offer similar benefits for insulin resistance; however, their effects are likely to be additive when combined. While individualization is important, resistance training’s role in enhancing insulin sensitivity makes option A the most accurate.

**15. What is the order of the four processes of motivational interviewing?**

1. Active listening, recall, focus, and planning.
2. Engage, focus, evoke, and plan.
3. Linking, evoking, focusing, and planning.
4. Active listening, focusing, encouraging, evoking, and planning.

**Correct answer: b**

Justification: The four processes of motivational interviewing, in order, are:

Engaging: Building rapport and trust with the patient.

Focusing: Defining and maintaining the direction of change.

Evoking: Eliciting the patient’s motivation for change.

Planning: Developing a collaborative strategy to achieve the desired change.
