# Supplementary material for: A Multifaceted Digital Intervention for the Prevention of Type 2 Diabetes Mellitus in Primary Care (PREDIABETEXT): Cluster Randomized Trial
Source: J Med Internet Res. 2025 Oct 9;27:e70981. doi: 10.2196/70981 (PMC12550449; doi:10.2196/70981)
Supplement: Multimedia Appendix 6 [file jmir_v27i1e70981_app6.docx]

Multimedia Appendix 6. Interview guides used in the within-trial process evaluation qualitative study.

1. Interview guide for healthcare professionals receiving online training PREDIABETEXT co-intervention

**INTRODUCTION**

- **Introduction of the researchers**
- **Overview of the interview plan** (objectives, duration, guidelines).
- **Brief presentation of the project**:
- We are conducting a research project with two components aimed at preventing diabetes development in at-risk individuals.
- On one hand, the project involves sending short-text messages (SMSs) to people at risk of developing diabetes, primarily focusing on healthy diet and physical activity.
- On the other hand, it includes an educational intervention for healthcare professionals (a training course on prediabetes).
- **Request for permission to audio-record the session**.

**INTERVIEW QUESTIONS**

**General**

- Do you find the course relevant, necessary, or useful?

**Format**

- Attendance: opinion on the online format.
- Content: videos, clinical cases, scientific articles, text documents, etc.
- Were the resources appropriate?
- Was the documentation provided sufficient?
- Videos: What is your opinion on them? Were they useful? Any specific aspects?
- Tasks: clinical cases, readings, video commentaries, etc.

**Course Duration**

- Was the duration appropriate?

**Evaluation Method**

- Pre- and post-training questionnaire: what is your opinion?

**Topics**

- Were there any topics that were too lengthy or difficult to follow?
- Were there any topics that required additional information?
- Were there any concepts or topics you felt were missing?

**Personal Opinion**

- Would you recommend this training to your colleagues?
- What aspects of this training would you improve?

**Acceptance and Implementation**

- Acceptance among healthcare professionals: What barriers and facilitators exist for the acceptance of this training among healthcare staff?
- Large-scale implementation: What barriers and facilitators do you foresee?

**SMS**

- Have any patients commented on or complained about the SMS intervention?
- Do you have any perceptions of its usefulness or patient satisfaction?

**CLOSURE (5 minutes)**

- End the session.
- Stop the recording.
- Thank the participants for their time.
- Ask if they would like to share their contact information (email) to stay updated on the project’s progress.

1. Interview guide for prediabetic patients receiving the SMS PREDIABETEXT co-intervention

Evaluation OF MESSAGE CONTENT

- **Themes**: Feedback on messages about healthy eating, physical activity, smoking, alcohol, etc.
- Were there any topics you particularly liked or found most interesting?
- Were there any missing information or aspects you would have liked to see included?
- Were there any messages you disliked or found offensive?
- Did you encounter any cultural conflicts? For instance, dietary advice that doesn’t align with your eating habits.
- Were the messages easy to understand?
- Was there any message you did not fully understand?

EXPLORATION OF MESSAGE CHARACTERISTICS

- What type of message tone did you like the most?
- Was there a specific message that caught your attention?
- What type of messages were most motivating in encouraging adherence to recommended behaviors (diet and/or exercise)?
- Was the frequency of the messages appropriate?
- What was your opinion about the timing of message delivery?
- Was there anything missing from the messages (e.g., more information, links, videos)?

EVALUATION OF USEFULNESS

- What is your overall opinion of the SMS intervention?
- Did you find the messages useful?
- What benefits did you derive from the messages?
- Did you learn anything new?
- Did the messages lead to any lifestyle changes?
- Did you refer back to the messages after receiving them?
- Did the messages increase your awareness of your condition?

SYSTEM APPLICABILITY

- Would you like to continue receiving these messages for a longer period?
- Would you recommend this program to others with blood sugar issues?

SUGGESTIONS FOR IMPROVEMENT

- What suggestions do you have to improve the system?
