# Supplementary material for: A Multifaceted Digital Intervention for the Prevention of Type 2 Diabetes Mellitus in Primary Care (PREDIABETEXT): Cluster Randomized Trial
Source: J Med Internet Res. 2025 Oct 9;27:e70981. doi: 10.2196/70981 (PMC12550449; doi:10.2196/70981)
Supplement: Multimedia Appendix 7 [file jmir_v27i1e70981_app7.docx]

Multimedia Appendix 7. Baseline demographic and clinical characteristics of the patients by the 3 trial arms.

|  |  | **Total (n=365)** | **Control (n=119)** | **Intervention A (SMS) (n=106)** | **Intervention B (SMS + training) (n=140)** |
| --- | --- | --- | --- | --- | --- |
| **Demographic characteristics** |  |  |  |  |  |
| Women, n (%) |  | 199 (54.5) | 69 (58.0) | 54 (50.9) | 76 (54.3) |
| Age (years), mean (SD) |  | 59.79 (9.75) | 60.75 (9.77) | 58.66 (9.60) | 59.84 (9.83) |
| Clinical characteristics, mean (SD) |  |  |  |  |  |
| Height (cm), mean (SD) |  | 162.82 (9.45) | 162.29 (9.70) | 163.28 (9.49) | 162.93 (9.24) |
| Weight (kg), mean (SD) |  | 84.38 (18.61) | 84.71 (21.73) | 84.57 (16.63) | 83.96 (17.22) |
| BMI (kg/m2), mean (SD) |  | 31.72 (5.97) | 31.98 (6.78) | 31.67 (5.56) | 31.54 (5.47) |
| WC (cm), mean (SD) |  | 103.77 (13.10) | 103.49 (15.77) | 103.92(11.48) | 103.89 (11.49) |
| HC (cm), mean (SD) |  | 110.06 (11.74) | 110.93 (13.76) | 109.35 (10.64) | 109.87 (10.66) |
| WHR, mean (SD) |  | 0.94 (0.08) | 0.93 (0.09) | 0.95 (0.07) | 0.94 (0.07) |
| SBP (mmHg), mean (SD) |  | 133.35 (15.50) | 130.78 (15.45) | 135.22 (16.42) | 134.10 (14.63) |
| DBP (mmHg), mean (SD) |  | 76.34 (10.80) | 74.60 (11.19) | 78.75 (11.04) | 75.97 (9.97) |
| FPG (mg/dl), mean (SD) |  | 104.28 (13.22) | 102.90 (12.20) | 105.18 (13.03) | 104.78 (14.16) |
| TG (mg/dl), mean (SD) |  | 141.09 (94.63) | 123.48 (54.17) | 156.85 (118.26) | 144.22 (99.79) |
| Chol (mg/dl), mean (SD) |  | 194.72 (38.53) | 195.98 (40.39) | 195.33 (34.78) | 193.14 (39.83) |
| LDL (mg/dl), mean (SD) |  | 117.68 (33.61) | 121.62 (35.62) | 116.88 (28.15) | 114.83 (35.41) |
| HDL (mg/dl), mean (SD) |  | 49.70 (11.60) | 50.22 (11.82) | 48.63 (11.27) | 50.07 (11.69) |
| TG/HDL, mean (SD) |  | 3.17 (3.58) | 2.65 (1.61) | 3.66 (3.67) | 3.25 (4.57) |
| Chol/HDL, mean (SD) |  | 3.97 (1.22) | 3.98 (1.03) | 4.16 (1.26) | 3.82 (1.31) |
| HbA1c (%), mean (SD) |  | 6.13 (0.16) | 6.13 (0.15) | 6.13 (0.15) | 6.15 (0.18) |
| Insulin level (U/ml), mean (SD) |  | 17.77(15.55) | 15.42(7.88) | 18.80 (13.7) | 18.76(20.87) |
| HOMA, mean (SD) |  | 4.86 (5.45) | 3.89 (2.26) | 4.87 (3.70) | 5.58 (7.76) |
| REGICOR-Framingham, mean (SD) |  | 4.04 (2.65) | 5.11 (4.30) | 3.98 (2.25) | 4.26 (2.95) |
| Categories of REGICOR-Framingham |  |  |  |  |  |
| Low risk, n (%) |  | 257 (72.4) | 85 (73.3) | 74 (70.5) | 98 (73.1) |
| Moderate or high risk, n (%) |  | 98 (27.6) | 31 (26.7) | 31 (29.5) | 36 (26.9) |
| **Adherence to Mediterranean diet** |  |  |  |  |  |
| MEDAS_score, mean (SD) |  | 7.52 (2.04) | 7.41 (2.08) | 7.56 (2.35) | 7.58 (1.74) |
| Low adherence, n (%) |  | 246 (67.4) | 83 (69.7) | 65 (61.3) | 98 (70.0) |
| Good adherence, n (%) |  | 119 (32.6) | 36 (30.3) | 41 (38.7) | 42 (30.0) |
| **Physical activity level** |  |  |  |  |  |
| Not very active, n (%) |  | 191 (52.4) | 69 (58.0) | 55 (51.9) | 67 (47.9) |
| Active, n (%) |  | 83 (22.7) | 22 (18.5) | 23 (21.7) | 38 (27.1) |
| Very active, n (%) |  | 91 (24.9) | 28 (23.5) | 28 (26.4) | 35 (25.0) |
| (METs minutes/week), mean (SD) |  | 1983.78 (2365.91) | 1987.94 (2413.43) | 1914.54 (2356.85) | 2032.66 (2347.65) |
| **Sedentary lifestyle** |  |  |  |  |  |
| Daily hours a day watching television, mean (SD) |  | 3.52 (1.78) | 3.64 (1.79) | 3.48 (1.96) | 3.44 (1.62) |
| Daily hours sitting in front of a computer/mobile/tablet screen, mean (SD) |  | 0.98 (1.55) | 1.18 (1.80) | 0.92 (1.42) | 0.85 (1.39) |
| Daily hours a day sitting in any means of transportation, mean (SD) |  | 0.33 (0.67) | 0.27 (0.36) | 0.29 (0.65) | 0.41 (0.85) |
| Daily hours a day sitting, mean (SD) |  | 4.57 (1.83) | 4.84 (1.82) | 4.45 (2.09) | 4.42 (1.61) |
| **Smoking habit** |  |  |  |  |  |
| Former or never smoker, n (%) |  | 293 (80.3) | 95 (79.8) | 93 (87.7) | 105 (75) |
| Current smoker, n (%) |  | 72 (19.7) | 24 (20.2) | 13 (12.3) | 35 (25) |
| **Alcohol use** |  |  |  |  |  |
| Alcohol units/week, mean (SD) |  | 3.03 (7.19) | 3.55 (7.48) | 3.01 (7.07) | 2.60 (7.04) |
| Audit |  |  |  |  |  |
| Low-risk consumption, n (%) |  | 346 (95.1) | 112 (94.1) | 100 (95.2) | 134 (95.7) |
| Hazardous or risky consumption, n (%) |  | 9 (5.9) | 7 (5.9) | 5 (4.8) | 6 (4.3) |

ALT: alanine aminotransferase, AST: aspartate aminotransferase, BMI: Body Mass Index, Chol: Cholesterol, CKD: Chronic Kidney Disease, Cr: Creatinine, DBP: Diastolic Blood Pressure, FIB-4: Fibrosis index based on the 4 factor, FPG: Fasting Plasma Glucose, GFR: Glomerular Filtration Rate, GGT: Gamma-glutamyl transpeptidase, HbA1c: Glycated hemoglobin, HC: Hip Circumference, HDL: High-Density Lipoprotein, HOMA: homeostasis model assessment, LDL: Low-Density Lipoprotein, MCH: mean corpuscular hemoglobin, MCHC: mean corpuscular hemoglobin concentration, MCV: Mean corpuscular volume, MPV: Mean Platelet Volume, PDW: Platelet Distribution Width, RDW: Red Cell Distribution Width, REGICOR-Framingham: Framingham-REgistre GIroní del COR, SBP: Systolic Blood Pressure, TG: Triglyceride, WC: Waist Circumference, WBC: white blood cell, WHR: Waist to Hip Ratio
