# Supplementary material for: A Multifaceted Digital Intervention for the Prevention of Type 2 Diabetes Mellitus in Primary Care (PREDIABETEXT): Cluster Randomized Trial
Source: J Med Internet Res. 2025 Oct 9;27:e70981. doi: 10.2196/70981 (PMC12550449; doi:10.2196/70981)
Supplement: Multimedia Appendix 8 [file jmir_v27i1e70981_app8.docx]

Multimedia Appendix 8. Clinical characteristics of intervention group A, intervention group B, and the control group at baseline and the 6-month time point.

| **Clinical characteristics** | **total pre-intervention (n=365)** | **total post-intervention (n=286)** | **Control pre-intervention (n=119)** | **Control post-intervention (n=92)** | **Intervention A (SMS) pre-intervention (n=106)** | **Intervention A (SMS) post-intervention (n=82)** | **Intervention B (SMS + training), pre-intervention (n=140)** | **Intervention B (SMS + training), post-intervention (n=112)** |
| --- | --- | --- | --- | --- | --- | --- | --- | --- |
| HbA1c (%), mean (SD) | 6.13 (0.16) | 6.12 (0.50) | 6.12 (0.15) | 6.18 (0.79) | 6.11 (0.15) | 6.07 (0.29) | 6.14 (0.18) | 6.12 (0.26) |
| Adherence to Mediterranean diet (score 0-14), mean (SD) | 7.51 (2.09) | 7.94 (1.95) | 7 (2.83) | 8.19 (2.17) | 7.56 (2.35) | 8.09 (1.87) | 7.54 (1.74) | 7.79 (1.99) |
| Total energy expenditure in physical activity (MET·min/week), mean (SD) | 2069.63 (2346.96) | 1941.99 (1927.11) | 2034.95 (1852.53) | 1299.49 (1405.63) | 2065.00 (2424.22) | 2084.15 (2217.80) | 2077.00 (2368.30) | 1925.89 (1740.26) |
| Sedentary lifestyle: NHS4 total, mean (SD) | 4.53 (1.84) | 4.52 (2.20) | 4.72 (1.70) | 4.34 (2.18) | 4.46 (2.15) | 4.54 (2.31) | 4.42 (1.69) | 4.66 (2.15) |
| Total alcohol units/week, mean (SD) | 2.95 (7.26) | 3.21 (5.79) | 3.12 (7.44) | 2.81 (4.38) | 2.85 (6.59) | 3.37 (6.12) | 3 (7.77) | 3.14 (5.75) |
| HOMA, mean (SD) | 4.86 (5.46) | 4.70 (4.17) | 3.88 (2.26) | 4.73 (5.12) | 4.87 (3.70) | 5.14 (4.00) | 5.58 (7.75) | 4.34 (3.25) |
| Weight (kg), mean (SD) | 84.38 (18.61) | 82.61 (17.30) | 84.71 (21.73) | 82.07 (20.05) | 84.57 (16.63) | 83.59 (16.85) | 83.95 (17.22) | 82.37 (15.17) |
| WC (cm), mean (SD) | 103.77 (13.09) | 102.64 (13.50) | 102.16 (14.91) | 102.16 (14.91) | 103.08 (13.36) | 103.08 (13.36) | 103.89 (11.48) | 102.71 (12.46) |
| HC (cm), mean (SD) | 110.06 (11.74) | 108.69 (11.10) | 110.93 (13.76) | 107.81 (12.17) | 109.34 (10.64) | 109.61 (11.10) | 109.86 (10.65) | 108.76 (10.18) |
| SBP (mmHg),  mean (SD) | 133.35 (15.50) | 135.18 (16.61) | 130.78 (15.45) | 134.97 (11.19) | 135.22 (16.42) | 133.41 (15.72) | 134.10 (14.62) | 136.58 (170.01) |
| DBP (mmHg), mean (SD) | 76.34 (10.80) | 77.70 (10.52) | 74.60 (11.19) | 76.68 (10.59) | 78.75 (11.04) | 78.87 (10.56) | 75.97 (9.97) | 77.71 (10.45) |
| FPG (mg/dl), mean (SD) | 104.28 (13.218) | 104.17 (20.06) | 102.90 (12.20) | 105.33 (28.16) | 105.18 (13.03) | 103.89 (16.02) | 104.78 (14.16) | 103.40 (13.82) |
| TG (mg/dl), mean (SD) | 141.09 (94.63) | 129.89 (70.08) | 123.48 (54.17) | 123.56 (69.49) | 156.85 (118.26) | 142.35 (81.87) | 144.22 (99.79) | 126.02 (59.95) |
| Total Chol (mg/dl), mean (SD) | 194.72 (38.53) | 193.68 (40.86) | 195.98 (40.39) | 192.31 (43.49) | 195.33 (34.785) | 201.49 (40.79) | 193.14 (39.83) | 189.10 (38.09) |
| LDL (mg/dl), mean (SD) | 194.72 (38.53) | 193.68 (40.86) | 195.98 (40.40) | 192.31 (43.49) | 195.33 (34.78) | 201.49 (40.79) | 193.14 (39.83) | 189.10 (38.09) |
| HDL (mg/dl), mean (SD) | 49.70 (11.60) | 51.79 (12.52) | 50.22 (11.84) | 50.44 (13.96) | 48.63 (11.27) | 50.96 (12.33) | 50.07 (11.69) | 51.85 (11.43) |
| Non-HDL Chol (mg/dl), mean (SD) | 145.17 (36.61) | 141.17 (40.45) | 146.06 (36.94) | 139.61 (43.10) | 146.98 (35.41) | 149.23 (42.43) | 143.01 (37.38) | 136.55 (35.92) |
| TG/HDL | 3.17 (3.58) | 2.80 (2.36) | 2.64 (1.61) | 2.64 (2.10) | 3.65 (3.67) | 3.08 (2.34) | 3.24 (4.57) | 2.73 (2.57) |
| Chol/HDL | 3.97 (1.22) | 3.89 (1.12) | 3.98 (1.03) | 3.84 (1.20) | 4.15 (1.26) | 4.14 (1.20) | 3.81 (1.26) | 3.74 (0.96) |
| GFR (ml/min/1.73 m2), mean (SD) | 87.12 (16.02) | 87.10 (15.45) | 85.05 (17.73) | 86.71 (15.46) | 88.26 (17.04) | 86.45 (19.01) | 88.10 (13.40) | 87.86 (12.50) |
| Serum Cr (mg/dl), mean (SD) | 0.84 (0.22) | 0.83 (0.21) | 0.85 (0.25) | 0.83 (0.24) | 0.84 (0.22) | 0.86 (0.24) | 0.82 (0.18) | 0.81 (0.16) |
| Urine Cr (mg/dl), mean (SD) | 109.22 (58.65) | 118.84 (62.08) | 111.60 (59.84) | 116.99 (61.65) | 109.59 (51.30) | 120.52 (61.59) | 106.87 (63.25) | 119.20 (63.34) |
| Urine microalbumin (mg/L), mean (SD) | 22.07 (52.08) | 20.18 (51.82) | 25.92 (61.65) | 20.98 (50.51) | 15.52 (39.60) | 18.06 (44.51) | 23.66 (51.47) | 21.05 (57.96) |
| Microalbumin/Cr | 27.49 (56.60) | 21.43 (48.60) | 29.85 (61.40) | 23.378 (63.70) | 23.01 (48.44) | 17.43 (28.63) | 28.81 (58.45) | 22.67 (43.92) |
| Insulin (µUI/ml), mean (SD) | 17.77 (15.55) | 18.22 (13.87) | 15.41 (7.88) | 18.08 (15.77) | 18.80 (13.07) | 19.69 (1.70) | 18.75 (20.86) | 17.25 (11.55) |
| FIB-4 , mean (SD) | 1.19 (0.63) | 1.26 (0.53) | 1.27 (0.860) | 1.31 (0.60 | 1.09 (0.44) | 1.15 (0.46) | 1.20 (0.47) | 1.28 (0.52) |
| Lipoprotein A, mean (SD) | 39.66 (50.89) | 41.74 (50.90) | 38.97 (47.11) | 41.23 (43.94) | 21.46 (30.53) | 34.74 (37.20) | 54.46 (61.88) | 47.54 (63.99) |
| AST (U/L),  mean(SD) | 23.23 (12.07) | 22.66 (9.18) | 23.84 (11.90) | 23.01 (9.71) | 22.30 (8.84) | 22.57 (6.71) | 23.40 (14.29) | 22.44 (10.27) |
| ALT (U/L),  mean (SD) | 26.95 (20.72) | 25.51 (17.54) | 26.81 (14.21) | 24.66 (15.20) | 25.71 (14.77) | 26.63 (12.68) | 28.05 (28.31) | 25.42 (21.93) |
| GGT(U/L),  mean (SD) | 43.94 (107.70) | 35.69 (38.75) | 55.25 (177.24) | 34.13 (30.01) | 39.53 (35.35) | 34.32 (23.64) | 37.36 (46.13) | 37.96 (51.78) |
| WBC (x10^9/L), mean (SD) | 7.48 (2.10) | 7.29 (2.03) | 7.69 (2.04) | 7.44 (2.01) | 7.60 (2.45) | 7.32 (2.30) | 7.22 (1.85) | 7.14 (1.85) |
| Neutrophil (x10^9/L), mean (SD) | 4.01 (1.58) | 3.94 (1.45) | 4.13 (1.41) | 4.12 (1.47) | 4.05 (1.78) | 3.86 (1.51) | 3.88 (1.55) | 3.85 (1.38) |
| Lymphocyte (x10^9/L), mean (SD) | 2.58 (0.84) | 2.46 (0.90) | 2.64 (0.79) | 2.45 (0.88) | 2.63 (1.02) | 2.51 (1.06) | 2.48 (0.72) | 2.42 (0.78) |
| Monocyte (x10^9/L), mean (SD) | 0.61 (0.21) | 0.60 (0.18) | 0.64 (0.22) | 0.60 (0.17) | 0.61 (0.21) | 0.59 (0.20) | 0.59 (0.19) | 0.60 (0.18) |
| Eosinophil (x10^9/L), mean (SD) | 0.23 (0.19) | 0.22 (0.22) | 0.22 (0.13) | 0.20 (0.12) | 0.26 (0.28) | 0.28 (0.37) | 0.22 (0.16) | 0.206 (0.10) |
| Basophil (x10^9/L), mean (SD) | 0.04 (0.03) | 0.05 (0.04) | 0.04 (0.03) | 0.05 (0.04) | 0.04 (0.03) | 0.05 (0.04) | 0.04 (0.03) | 0.05 (0.04) |
| Hematite (x10^12/L), mean (SD) | 4.77 (0.42) | 4.72 (0.41) | 4.75 (0.42) | 4.71 (0.43) | 4.77 (0.40) | 4.729 (0.36) | 4.80 (0.43) | 4.73 (0.43) |
| Hemoglobin(g/dl), mean (SD) | 14.35 (1.24) | 14.48 (1.18) | 14.30 (1.23) | 14.51 (1.30) | 14.30 (1.33) | 14.44 (1.10) | 14.43 (1.17) | 14.48 (1.13) |
| Haematocrit (%), mean (SD) | 43.67 (3.66) | 43.48 (3.50) | 43.60 (3.81) | 43.52 (3.85) | 43.46 (3.84) | 43.37 (3.08) | 43.88 (3.39) | 43.52 (3.53) |
| MCV (fl), mean (SD) | 91.57 (5.34) | 92.19 (5.15) | 91.90 (4.70) | 92.53 (4.71) | 91.20 (5.64) | 91.88 (5.33) | 91.57 (5.63) | 92.143 (5.40) |
| MCH (pg), mean (SD) | 30.10 (1.93) | 30.70 (1.85) | 30.15 (1.66) | 30.84 (1.69) | 30.03 (2.09) | 30.61 (2.00) | 30.11 (2.03) | 30.66 (1.87) |
| MCHC(g/dl), mean (SD) | 32.87 (0.90) | 33.31 (1.02) | 32.81 (0.93) | 33.33 (1.11) | 32.92 (0.97) | 33.30 (1.02) | 32.88 (0.83) | 33.29 (0.94) |
| RDW (%), mean (SD) | 12.78 (1.02) | 13.37 (0.88) | 12.85 (0.92) | 13.48 (0.84) | 12.68 (1.11) | 13.21 (0.93) | 12.80 (1.02) | 13.39 (0.86) |
| Plaquette (x10^9/L), mean (SD) | 254.19 (65.29) | 247.33 (66.80) | 252.42 (63.27) | 248.41 (70.15) | 260.78 (66.50) | 252.90 (66.55) | 250.70 (66.15) | 241.62 (64.19) |
| MPV (fl), mean (SD) | 8.60 (1.18) | 10.25 (1.29) | 8.72 (1.23) | 10.34 (1.30) | 8.50 (1.11) | 10.15 (1.09) | 8.56 (1.19) | 10.267 (1.42) |
| PDW (%), mean (SD) | 14.69 (1.37) | 13.75 (0.83) | 13.69 (0.73) | 14.64 (1.39) | 14.77 (1.36) | 13.64 (0.78) | 14.68 (1.37) | 13.91 (0.86) |
| **CKD Prognosis, N (%)** |  |  |  |  |  |  |  |  |
| Normal | 15 (7.3) | 16 (6) | 5 (7.4) | 4 (4.5) | 2 (3.4) | 5 (6.7) | 8 (10.3) | 7 (6.8) |
| Moderate | 6 (2.9) | 2 (0.8) | 3 (4.4) | 1 (1.1) | 1 (1.7) | 0 | 2 (2.6) | 1 (1) |
| High | 184 (89.8) | 248 (93.2) | 60 (88.2) | 83 (94.3) | 56 (94.9) | 70 (93.3) | 68 (87.2) | 95 (92.2) |
| **Physicalactivitylevel, N (%)** |  |  |  |  |  |  |  |  |
| Notvery active | 191 (52.3) | 143 (47.4) | 69 (58) | 53 (54.6) | 55 (51.9) | 38 (43.2) | 67 (47.9) | 52 (44.4) |
| Active | 83 (22.7) | 66 (21.9) | 22 (18.5) | 21 (21.6) | 23 (21.7) | 20 (22.7) | 38 (27.1) | 25 (21.4) |
| Very active | 91 (24.9) | 93 (30.8) | 28 (23.5) | 23 (23.7) | 28 (26.4) | 30 (34.1) | 35 (25) | 40 (34.2) |
| **Smoking status, N (%)** |  |  |  |  |  |  |  |  |
| Neversmoker | 163 (44.7) | 154 (51) | 52 (43.7) | 47 (48.5) | 47 (44.3) | 47 (53.4) | 64 (45.7) | 60 (51.3) |
| Formersmoker | 130 (35.6) | 95 (31.5) | 43 (36.1) | 32 (33) | 46 (43.4) | 31 (35.2) | 41 (29.3) | 32 (27.4) |
| Non dailysmoker | 5 (1.4) | 6 (2) | 2 (1.7) | 3 (3.1) | 0 | 2 (2.3) | 3 (2.1) | 1 (0.9) |
| Dailysmoker | 67 (18.4) | 47 (15.6) | 22 (18.5) | 15 (15.5) | 13 (12.3) | 8 (9.1) | 32 (22.9) | 24 (20.5) |
| **Level of motivation/capacity for change, N (%)** |  |  |  |  |  |  |  |  |
| Low | 136 (59.4) | 67 (38.3) | 35 (50) | 22 (41.5) | 41 (65.1) | 20 (35.1) | 60 (62.5) | 25 (38.5) |
| High | 93 (40.6) | 108 (61.7) | 35 (50) | 31 (58.5) | 22 (34.9) | 37 (64.9) | 36 (37.5) | 40 (61.5) |

^ALT: alanine aminotransferase, AST: aspartate aminotransferase, BMI: Body Mass Index, Chol: Cholesterol, CKD: Chronic Kidney Disease, Cr: Creatinine, DBP: Diastolic Blood Pressure, FIB-4: Fibrosis index based on the 4 factor, GFR: Glomerular Filtration Rate, GGT: Gamma-glutamyl transpeptidase, HbA1c: Glycated haemoglobin, HC: Hip Circumference, HDL: High-Density Lipoprotein, HOMA: homeostasis model assessment, LDL: Low-Density Lipoprotein, MCH: mean corpuscular hemoglobin, MCHC: mean corpuscular hemoglobin concentration, MCV: Mean corpuscular volume, MPV: Mean Platelet Volume, PDW: Platelet Distribution Width, RDW: Red Cell Distribution Width, SBP: Systolic Blood Pressure, TG: Triglyceride, WC: Waist Circumference, WBC: white blood cell, WHR: Waist to Hip Ratio^
