# Supplementary material for: A Multifaceted Digital Intervention for the Prevention of Type 2 Diabetes Mellitus in Primary Care (PREDIABETEXT): Cluster Randomized Trial
Source: J Med Internet Res. 2025 Oct 9;27:e70981. doi: 10.2196/70981 (PMC12550449; doi:10.2196/70981)
Supplement: Multimedia Appendix 9 [file jmir_v27i1e70981_app9.docx]

Multimedia Appendix 9. Effects of the digital intervention targeted at patients (intervention group A) and the combined digital intervention targeted at patients and health care professionals (intervention group B) on clinical, physiological, and behavioral outcomes (per-protocol analysis).

| **Variable** |  | **Per protocol analysis** | | | | |  |
| --- | --- | --- | --- | --- | --- | --- | --- |
|  | **Control** | **Intervention A** |  |  | **Intervention B** |  |  |
|  | **Mean (SD)** | **Mean (SD)** | **Beta (95% CI)** | ***P* value** | **Mean (SD)** | **Beta (95% CI)** | ***P* value** |
|  |  |  | **Intervention A *vs.* control** |  |  | **Intervention B *vs.* control** |  |
| **Glycemic control** |  |  |  |  |  |  |  |
| **HbA1c^a^** | 6.18 (0.79) | 6.07 (0.29) | -0.10 (-0.27 to 0.07) | 0.25 | 6.12 (0.25) | -0.07 (-0.23 to 0.09) | 0.39 |
| **FPG^b^** | 105.33 (28.15) | 103.89 (16.02) | -3.32 (-9.67 to 3.18) | 0.32 | 103.40 (13.82) | -3.42 (-9.26 to 2.43) | 0.25 |
| **Insulin^c^** | 18.08 (15.76) | 19.69 (1.69) | -1.280 (-7.10 to 4.54) | 0.66 | 17.25 (11.54) | -4.21 (-8.20 to -0.21) | 0.03 |
| **HOMA^d^** | 4.73 (5.11) | 5.14 (4) | -0.538 (-2.17 to 1.09) | 0.51 | 4.34 (3.25) | -1.57 (-2.78 to -0.35) | 0.01 |
| **Lipid profile** |  |  |  |  |  |  |  |
| **TG^a^** | 123.56 (69.49) | 142.35 (81.87) | 3.868 (-17.04 to 24.78) | 0.71 | 126.02 (59.95) | -6.18 (-20.52 to 8.15) | 0.39 |
| **Total cholesterol**^a^ | 192.31 (43.49) | 201.49 (40.79) | 8.98 (-1.11 to 19.08) | 0.08 | 189.10 (38.09) | -0.14 (-8.91 to 8.61) | 0.97 |
| **LDL**^c^ | 114.92 (38.18) | 122.30 (33.42) | 11.28 (2.41 to 20.15) | 0.01 | 112.81 (33.34) | 2.80 (-5.35 to 10.96) | 0.50 |
| **HDL**^d^ | 50.44 (13.96) | 50.96 (12.32) | -0.22 (-2.75 to 2.29) | 0.86 | 51.85 (11.42) | -0.33 (-2.61 to 1.95) | 0.77 |
| **Anthropometric outcomes** |  |  |  |  |  |  |  |
| **Weight^a^** | 82.07 (20.05) | 83.58 (16.85) | -0.24 (-1.64 to 1.15) | 0.73 | 82.36 (15.16) | -0.34 (-1.40 to 0.72) | 0.52 |
| **Waist^b^** | 102.16 (14.91) | 103.08 (13.36) | -0.51 (-2.38 to 1.35) | 0.59 | 102.71 (12.46) | -0.78 (-2.67 to 1.10) | 0.41 |
| **Hip^c^** | 107.81 (12.17) | 109.61 (11.10) | 1.59 (-0.03 to 3.22) | 0.055 | 108.76 (10.18) | 0.43 (-1.01 to 1.87) | 0.55 |
| **BMI^d^** | 30.67 (5.76) | 31.25 (5.59) | -0.03 (-0.59 to 0.52) | 0.89 | 31.06 (4.89) | -0.12 (-0.56 to 0.31) | 0.58 |
| **Cardiovascular outcomes** |  |  |  |  |  |  |  |
| **REGICOR-Framingham ^a^** | 5.82 (3.34) | 5.50 (3.23) | -0.008 (-0.69 to 0.67) | 0.98 | 6.8 (2.91) | 0.05 (-0.54 to 0.65) | 0.85 |
| **SBP^b^** | 134.97 (11.19) | 133.41 (15.72) | -3.66 (-7.58 to 0.26) | 0.06 | 136.58 (170.01) | -1.21 (-4.92 to 2.49) | 0.52 |
| **DBP^c^** | 76.68 (10.59) | 78.87 (10.56) | -0.20 (-2.80 to 2.38) | 0.87 | 77.71 (10.45) | 0.09 (-2.29 to 2.47) | 0.93 |
| **Lifestyle outcomes** |  |  |  |  |  |  |  |
| **Adherence to Mediterranean diet (score 0-14)^a^** | 8.19 (2.167) | 8.09 (1.875) | 0.55 (-0.02 to 1.12) | 0.06 | 7.79 (1.988) | 0.27 (-0.28 to 0.82) | 0.33 |
| **Total energy expenditure in physical activity (MET·min/week)^b^** | 1299.49 (1405.62) | 2084.15 (2217.79) | 311.97 (-328.98 to 952.93) | 0.34 | 1925.89 (1740.26) | 102.21 (-427.02 to 631.45) | 0.70 |
| **Sedentary lifestyle: NHS4Total^c^** | 4.34 (2.17) | 4.54 (2.31) | 0.32 (-0.26 to 0.92) | 0.28 | 4.66 (2.15) | 0.46 (-0.07 to 0.99) | 0.08 |
| **Total alcohol units/week^d^** | 2.81 (4.38) | 3.37 (6.11) | 1.02 (-0.39 to 2.45) | 0.15 | 3.14 (5.75) | 0.88 (-0.40 to 2.16) | 0.17 |

^a^Adjusted for baseline HbA1c. ^b^Adjusted for baseline FPG. ^c^Adjusted for baseline Insulin. ^d^Adjusted for baseline HOMA

BMI: Body Mass Index, Chol: Cholesterol, DBP: Diastolic Blood Pressure, FPG: fasting plasma glucose, HbA1c: Glycated haemoglobin, HC: Hip Circumference, HDL: High-Density Lipoprotein, HOMA: homeostasis model assessment, LDL: Low-Density Lipoprotein, REGICOR-Framingham: Framingham-REgistre GIroní del COR, SBP: Systolic Blood Pressure, TG: Triglyceride, WC: Waist Circumference, WHR: Waist to Hip Ratio
