# Supplementary material for: A Multifaceted Digital Intervention for the Prevention of Type 2 Diabetes Mellitus in Primary Care (PREDIABETEXT): Cluster Randomized Trial
Source: J Med Internet Res. 2025 Oct 9;27:e70981. doi: 10.2196/70981 (PMC12550449; doi:10.2196/70981)
Supplement: Multimedia Appendix 10 [file jmir_v27i1e70981_app10.docx]

Multimedia Appendix 10. Risk of progression to type 2 diabetes at the 6-month time point.

| **Risk Category** | **Control (n = 119)** | **Intervention A (n = 106)** | **Intervention B (n = 140)** |
| --- | --- | --- | --- |
| **Low** | 87 (73.1%) | 75 (70.8%) | 102 (72.9%) |
| **Medium** | 24 (20.2%) | 21 (19.8%) | 26 (18.6%) |
| **High** | 8 (6.7%) | 10 (9.4%) | 12 (8.6%) |

Risk categories are defined as: low = FPG 100–125 mg/dL only; medium = HbA1c 6.0–6.4% only; and high = both criteria.
