# Supplementary material for: A Multifaceted Digital Intervention for the Prevention of Type 2 Diabetes Mellitus in Primary Care (PREDIABETEXT): Cluster Randomized Trial
Source: J Med Internet Res. 2025 Oct 9;27:e70981. doi: 10.2196/70981 (PMC12550449; doi:10.2196/70981)
Supplement: Multimedia Appendix 11 [file jmir_v27i1e70981_app11.docx]

Multimedia Appendix 11. Health care professionals’ qualification scores (0-15) before and after the training program and at the 6-month time point.

|  | **n** | **Pre-intervention, mean (SD)** | **Post-intervention, mean (SD)** | **6 months follow-up, mean (SD)** | **Chi-Square** | **df** | **P value** |
| --- | --- | --- | --- | --- | --- | --- | --- |
| Control group qualification score (0-15) | 11 | Not assessed | Not assessed | 7.09 (1.37) | N/A | N/A | N/A |
| Intervention group A qualification score (0-15) | 12 | Not assessed | Not assessed | 6.33 (2.06) | N/A | N/A | N/A |
| Intervention group B qualification score (0-15) | 18 | 6.83 (1.33) | 12.17 (2.20) | 8.89 (3.92) | 12.76 | 2 | 0.002^*^ |
| Total qualification score (0-15) | 41 | N/A | N/A | 7.66 (3.07) | N/A | N/A | N/A |

^*^Friedman test, comparison of the effect of educational intervention on group 3
